# Role of cancer-associated fibroblast-derived exosomes in pancreatic cancer: clinical therapeutic potential and targeting challenges

**DOI:** 10.3389/fimmu.2026.1805988

**Published:** 2026-04-22

**Authors:** Weibo Hong

**Affiliations:** The Third Affiliated Hospital, Southern Medical University, Guangzhou, Guangdong, China

**Keywords:** biomarkers, cancer-associated fibroblast-derived exosomes, clinical implications, exosomes, pancreatic cancer, target delivery

## Abstract

Pancreatic cancer (PC)’s lethality is determined by late diagnosis and treatment resistance. The tumor microenvironment (TME), driven by cancer-associated fibroblasts (CAFs), is a crucial contributor. Within the TME, multiple cell types, including cancer cells, stromal cells, and immune cells, produce exosomes (EXOs), which are nanoscale extracellular vesicles (EVs) that mediate intercellular communication. Among these, CAF-derived EXOs (CAF-EXOs) conduct crucial interactions with cancer cells, conveying molecular cargo that promotes tumor proliferation, invasion, metastasis, metabolic reprogramming, and chemoresistance. Because CAF-EXOs provide a source of sensitive blood-based biomarkers for early detection and represent potential therapeutic targets whose disruption might overcome stromal-driven resistance, research on CAF-EXOs is crucial for addressing the fundamental shortcomings of current PC therapy. This narrative review synthesizes the critical significance of CAF-EXOs as master regulators of PC development and therapeutic resistance. We reveal how these EVs convey specialized molecular cargo, including proteins, lipids, and non-coding RNAs. By identifying CAF-EXOs as essential mediators of chemoresistance and stromal immunosuppression, we emphasize their dual potential as attractive liquid biopsy biomarkers and therapeutic targets. The study concludes with a critical examination of the translational landscape, reviewing new strategies to target exosomal biogenesis, uptake, or cargo, while also noting the enormous biological and technological challenges that must be overcome to achieve therapeutic relevance. Ultimately, altering the CAF-EXO communication axis provides a new avenue to overcome stromal-driven resistance and improve outcomes in PC.

## Graphical Abstract

**Figure:**
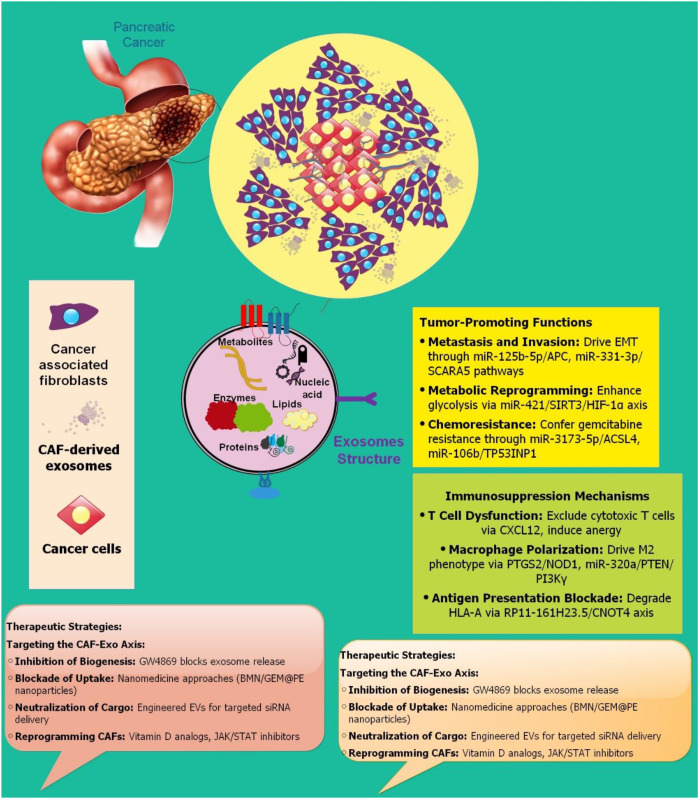

## 1 Introductions

PC is a highly fatal tumor, where early identification dramatically increases survival. However, most patients are discovered at an advanced stage owing to a lack of early symptoms, leaving fewer than 20% eligible for curative surgery and resulting in a poor 5-year survival rate of just 13% (1–3). Current diagnostic approaches, which combine imaging (computed tomography/magnetic resonance imaging) and invasive tissue biopsy (endoscopic ultrasound-guided fine-needle aspiration/biopsy), lack sufficient sensitivity for early-stage disease and entail procedural risks. For the early identification of PC, there is a critical unmet clinical need for precise, minimally invasive, and affordable techniques (1, 2).

Additionally, surgery is the sole treatment option for pancreatic ductal adenocarcinoma (PDAC), a very deadly malignancy. However, most patients are diagnosed at an advanced, inoperable stage (4–6). Even among individuals who have surgery, over 80% develop a recurrence within five years, typically caused by undiscovered micrometastases at the time of resection. Current chemotherapy treatments give only a moderate survival advantage, and relapses are prevalent, leading to an overall 5-year survival rate of approximately 13%. This underscores the urgency of new strategies. A greater understanding of the molecular processes, signaling pathways, and risk factors driving PDAC is crucial for identifying novel therapeutic targets, developing prognostic indicators, and developing tailored prevention and early intervention strategies (4, 5).

Carcinogenesis is a dynamic process involving diverse cells. Communication occurs not only between cancer cells but also with the tumor microenvironment (TME), which includes stromal cells, such as fibroblasts and immune cells (7, 8). New approaches focus on the TME, whereas earlier treatments targeted cancer cells directly. Although they help only a small percentage of patients, immunotherapies such as checkpoint inhibitors show promise. Trials have shown better results when they are used with anti-angiogenic medications (9). Cancer-associated fibroblasts (CAFs) are a significant stromal component, notably in PC, where they form most of the tumor mass. CAFs are varied; specific subtypes induce cancer, while others may restrict development. When CAFs are activated near tumors, they alter the extracellular matrix (ECM) and promote invasion, immune evasion, and treatment resistance by secreting cytokines and exosomes (EXOs) (9).

The translation of EXO-based diagnostics and therapeutics for PC faces significant translational challenges. The high cost and technical complexity of separating high-purity, PDAC-specific EXOs from patient samples are substantial obstacles. Because modifications to the delivery of medications, such as small interfering RNA (siRNA), must not unintentionally accelerate tumor growth, the safety of modified EXOs remains a considerable concern. Furthermore, manufacturing procedures must be standardized to produce consistent, contaminant-free EXO preparations that meet clinical requirements. Despite these challenges, EXOs have enormous promise as natural nanocarriers for tailored drug delivery to overcome therapeutic resistance and as non-invasive liquid biopsy instruments for early detection and staging. Overcoming current limitations in isolation, engineering, and scalable production through thorough clinical research is essential to realize their potential as precise tools for PDAC care (10).

In PDAC, CAF-derived EXOs (CAF-EXOs) are identified as key mediators of the malignant phenotype. Pro-tumorigenic pathways, including Phosphoinositide 3-Kinase/Protein Kinase B (PI3K/Akt), transforming growth factor beta (TGF-β), and STAT, are activated when their cargo—which includes specific microRNAs (miRNAs), long non-coding RNAs (lncRNAs), and proteins—is transported to cancer and other stromal cells. They change the ECM to enable invasion, dampen anti-tumor immune responses by training immune cells, and confer chemoresistance by modifying drug absorption and cancer cell survival pathways. This presents CAF-EXOs not only as essential drivers of disease but also as prospective sources for liquid biopsies and potential therapeutic targets (11–13).

Therapeutically, options include directly reducing EXO biogenesis/release, limiting their uptake by recipient cells, or depleting specific oncogenic cargos. However, addressing CAF-EXOs poses significant challenges. These include the biological challenge of achieving targeted delivery without disrupting homeostatic EXO activity in normal tissues, the physical obstacles posed by the PDAC stroma to drug administration, and the variety of CAF populations and their exosomal payloads. Overcoming these difficulties requires new delivery strategies and a more comprehensive molecular understanding of CAF-EXO biology (12, 14–16).

This narrative review provides a comprehensive synthesis of how CAF-EXOs regulate the malignant progression and treatment resistance of PC. By outlining their involvement in promoting proliferation, metastasis, immunological suppression, and chemoresistance via the transport of unique molecular cargo, we define CAF-EXOs as significant therapeutic targets. The study closes with a critical examination of the translational environment, assessing new ways to harness this exosomal communication axis for diagnosis and treatment, and pinpointing the significant conceptual and practical challenges that lie on the route to therapeutic effect.

## 2 Cancer-associated fibroblasts in pancreatic cancer

CAFs form a large cell population in the TME and play a dominant role in the stromal cell–cell interaction hierarchy. Quiescent tissue-resident fibroblasts are the primary source of CAFs, which are constantly activated and have increased synthetic, metabolic, and proliferative capacity (17, 18). This activation is typically initiated by inflammatory mediators and soluble factors, such as TGF-β and interleukin (IL)-1, released by tumor cells and other TME components. Activated CAFs perform many activities, including modifying the ECM, secreting growth factors, cytokines, and chemokines, promoting angiogenesis, and inducing immune evasion, which make them crucial for tumor survival, proliferation, and metastasis (19, 20).

According to research, CAFs can originate from a variety of sources, including pre-existing quiescent stellate cells, normal fibroblasts (NFs), fibroblasts, bone marrow-derived mesenchymal stem cells (MSCs), endothelial cells, epithelial cells, pericytes, smooth muscle cells, and adipocytes. Based on the expression of specific markers, classifications of CAFs primarily focus on three basic subtypes: myofibroblastic CAFs (myCAFs), inflammatory CAFs (iCAFs), and antigen-presenting CAFs (apCAFs). These subtypes undergo modifications during tumor growth and are controlled at the spatial level. For instance, in PC, three distinct CAF subtypes coexist and display varied functional properties and transcriptional plasticity. The actions of myCAFs and iCAFs depend on the production of ECM and immunomodulatory molecules, respectively, whereas apCAFs interact directly with T cells to increase T cell fatigue (20, 21).

Through mechanisms including TGF-β signaling and the transmission of resistance mediators (such as via EXOs) to cancer cells, CAFs play a crucial role in therapeutic resistance (20). In addition, CAFs contribute to immune evasion by modulating dendritic cell (DC) function, thereby impairing effective anti-tumor immune responses. Particularly in stroma-rich malignancies like PDAC, targeting these CAF-DC interactions—for example, by blocking WNT2—represents a viable approach to overcome immune resistance (20, 22–24).

CAFs have a well-established role in promoting cancer progression by supporting proliferation, invasion, and therapeutic resistance. In PC, for example, they guide invasion and may metastasize to distant sites. Preclinical models demonstrate that targeting CAFs can enhance chemotherapeutic delivery and response. Nevertheless, this approach has proved difficult (25–27). More aggressive and metastatic illness has sometimes resulted from clinical and experimental attempts to reduce or suppress CAFs, such as using hedgehog inhibitors. This shows that specific CAF populations may initially help control malignancies. This functional duality is crucial. Distinct CAF subtypes exist, with some promoting cancer and others inhibiting it. For example, a tumor-restraining function is linked to Meflin-positive CAFs. Retinoids and other medications can convert tumor-promoting CAFs into a more restrained, quiescent state; this approach is now undergoing clinical trials. The tumor-suppressive strategies of CAFs may include establishing a restrictive ECM and regulating immune cells. The timing and specificity of any therapeutic intervention are crucial, since the balance of CAF subtypes and their activities appears to be geographically and temporally regulated as the tumor develops (27).

Researchers examined blood levels of cellular fibronectin (C-FN) and its sialylated form (S-FN) in patients with PDAC. The data demonstrated a key relationship between these stromal markers, the TME, and clinical outcomes. Patients who were blood S-FN-negative revealed a high presence of vimentin-positive CAFs in the stroma, extensive fibrous development, poor response to chemoradiotherapy, and a terrible prognosis. S-FN-positive subjects, on the other hand, showed modest therapeutic effects and had fewer CAFs. Furthermore, in advanced metastatic cases, a large proportion of PDAC cells and surrounding CAFs expressed the epithelial-mesenchymal transition (EMT)-related factor CD44, suggesting cross-talk that may facilitate early distant metastasis. The results indicate that decreased autocrine fibronectin (S-FN negativity) is associated with a CAF-rich, therapy-resistant stroma driven by EMT (28).

According to studies, CAFs in glutamine-deficient pancreatic tumors depend on macropinocytosis to maintain a myCAF phenotype that encourages a thick, fibrotic, and immunosuppressive stroma. However, blocking macropinocytosis impairs this metabolic adaptation, unleashing an intrinsic MEK-ERK-driven inflammatory program that changes myCAFs into iCAFs. This metabolic reprogramming and CAF subtype shift successfully remodel the TME by reducing collagen deposition, widening blood vessels, and increasing T cell infiltration. As a result, this stromal remodeling makes ordinarily resistant PCs more susceptible to chemotherapy and immunotherapy. According to the research, macropinocytosis is a potential therapeutic target to overcome stromal barriers in PC and a metabolic regulator of the CAF landscape (29).

PDAC microenvironment orchestrators driving development, immune evasion, and severe therapeutic resistance include CAFs, which are dominant and functionally diverse. Recent research has shown a significant drawback, though: CAFs may not always promote tumor growth. This is demonstrated by the paradoxical worsening of illness after widespread CAF depletion and by the discovery of specific CAF subtypes that inhibit tumor growth, such as Meflin-positive CAFs. A big knowledge gap is the inability to target specific, pathological CAF subpopulations without disrupting potentially beneficial stromal functions. This is due to metabolic plasticity, which includes reliance on micropinocytosis and dynamic cross-talk via markers like S-FN and CD-44. The need to go beyond static categorization is demonstrated by a comprehensive study showing that basic CAF targeting has been clinically ineffective. To fill these gaps, EXO analysis is becoming increasingly important. This is because TAF-EXOs, derived from tumor-associated fibrosis, carry specific molecular cargo, including miRNAs, lncRNAs, and proteins. These cargoes directly mediate the communication between tumors, stroma, and the immune system, as well as resistance. Additionally, TAF-EXOs provide a living, breathing picture of the changing CAF landscape, reflecting results from liquid biopsies, enabling more accurate diagnostic and treatment approaches.

### 2.1 Biogenesis and composition of cancer-associated fibroblast-derived exosomes

Extracellular vesicles (EVs) are phospholipid bilayer particles produced by virtually all cells, containing a variety of molecular payloads (nucleic acids, proteins, lipids) and present in physiological fluids such as blood. They are generically categorized into EXOs, microvesicles, and apoptotic bodies. EXOs, spanning 30–150 nm, originate from the endosomal pathway: they develop as intraluminal vesicles (ILVs) inside multivesicular bodies (MVBs) via processes that rely on the Endosomal Sorting Complex Required for Transport (ESCRT) complex or ESCRT-independent pathways involving tetraspanins or ceramides. These MVBs may be destroyed by lysosomes or, driven by Rab GTPases, merge with the plasma membrane to release their ILVs as EXOs into the extracellular environment (30–32).

EXO biogenesis is primarily directed by the ESCRT machinery, which is essential for generating ILVs inside MVBs. The process begins when ESCRT-0 identifies ubiquitinated cargo proteins on the endosomal membrane. After that, ESCRT-I and II help assemble ESCRT-III, which is driven by the ATPase Vps4, thereby causing membrane budding and scission to generate ILVs. While the direct regulatory function of ESCRT in EXO release remains contested, critical ESCRT-associated proteins, such as Alix, are frequently identified in EXOs and participate in cargo selection (33–37). In early endosomes, endocytosed cargo is sorted concurrently. Cargo intended for secretion enters the endosomal maturation pathway, where it is sorted into late endosomes/MVBs. These MVBs may either fuse with lysosomes for destruction or with the plasma membrane to release ILVs as EXOs. This maturation involves dynamic membrane remodeling, including the replacement of Rab5 with Rab11 and the concentration of ceramides—a process aided by the ceramide transfer protein and controlled by GTPases such as Rab31. These ESCRT-dependent and lipid-driven processes combined enable the precise packing and release of exosomal cargo (35, 38–42).

**Figure 1:**
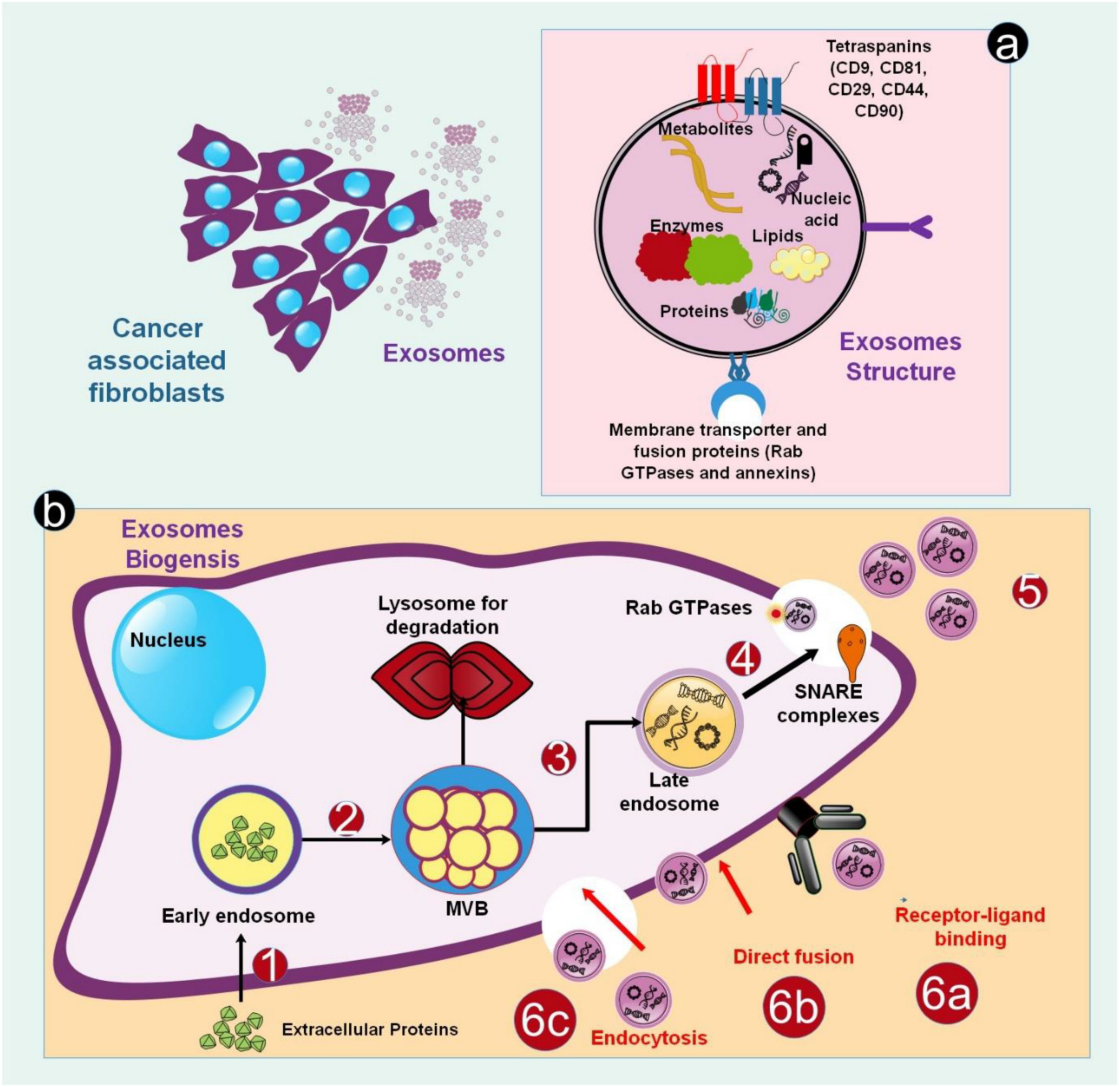
CAF-EXOs biogenesis and structure. **(a)** CAF-EXOs are phospholipid bilayers derived from the plasma membrane that include parent cell cytoplasm. In terms of proteins, lipids, and miRNAs, a significant percentage of EXOs show homology. **(b)** EXO biogenesis and the pathways via which they enter the target cell. (1-2) The biogenesis of EXOs starts in the endosomal system. Within the endosomal system, internalized loads are contained in early endosomes, which mature into late endosomes or MVB. Many chemicals are also transferred from the trans-Golgi network and perhaps from the cytoplasm. Additionally, MVBs may be transported to lysosomes for destruction or travel along microtubules to fuse with the cell membrane and release EXOs into the extracellular environment. (3-5) Several crucial elements, including SNARE complexes and Rab GTPases, are necessary for the exact mechanism of MVB fusion with the membrane. (6) EXOs use three key tactics to affect target cells: (6a) signal amplification mediated by recipient cell receptors; (6b) direct attachment to the plasma membrane and fusion; and (6c) endocytosis (44, 49, 50).

CAF-EXOs carry a diverse and potent molecular payload that promotes tumor development. Sonic Hedgehog (SHH) and CD81, two of their protein components, stimulate essential signaling pathways in recipient cancer cells, including the Wnt/β-catenin and Hedgehog pathways, to promote growth and metastasis. They also carry functional non-coding RNAs (ncRNAs), including miRNAs and lncRNAs, which reduce target gene expression and thereby impact tumor formation. Additionally, these EXOs transport vital metabolites, including lipids, TCA cycle intermediates, and amino acids (such as glutamine), which directly support cancer cells’ metabolic reprogramming, enabling their growth, invasion, and resistance to treatment (21, 43–48) (**Figure 1**).

## 3 Multifunctional roles of CAF-EXOs in pancreatic cancer pathogenesis

**Figure 2:**
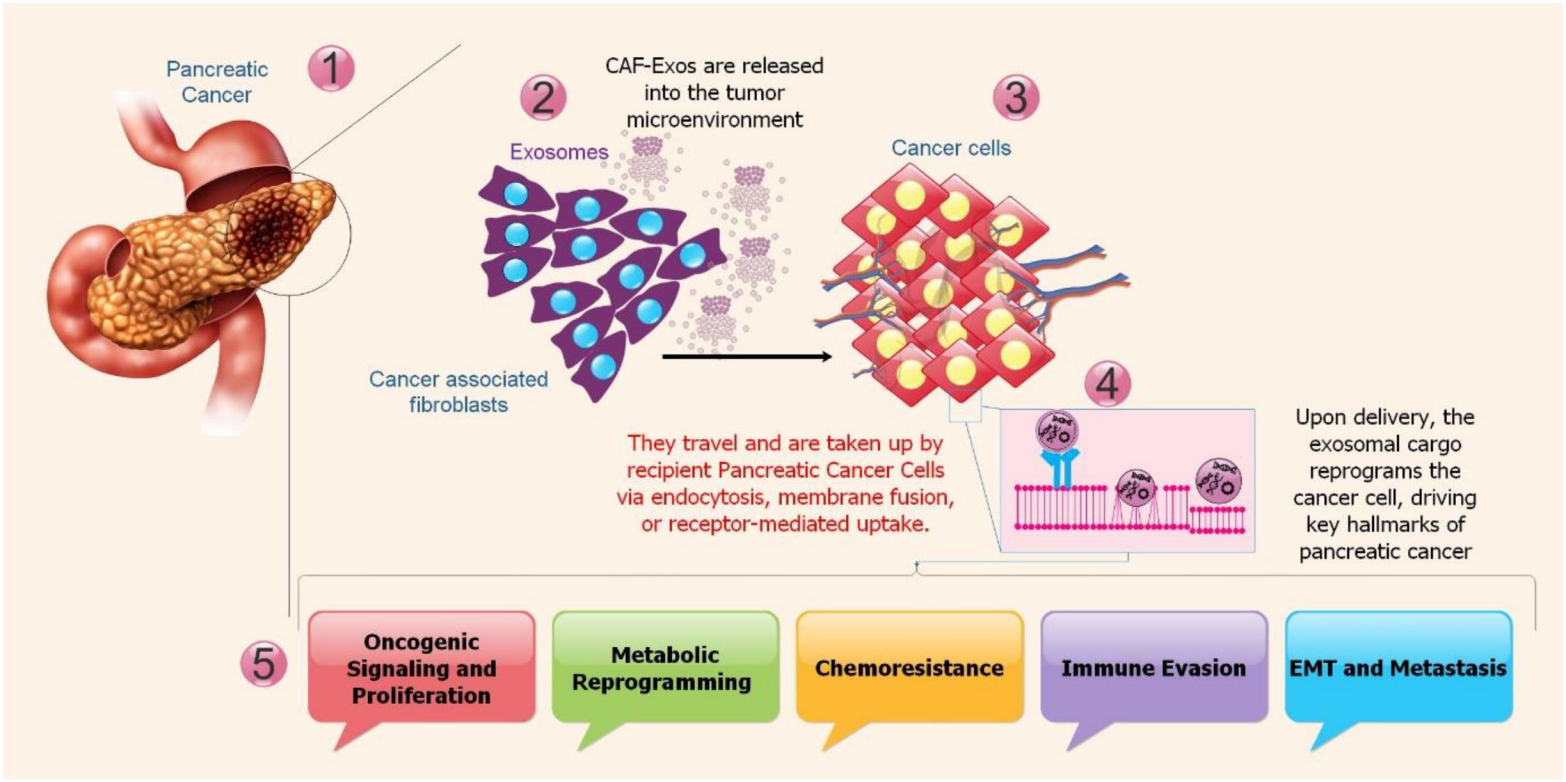
The functional effect of EXOs produced from CAF on PC cells and their intercellular transmission. (1-3) After escaping into the TME, EXOs produced by CAFs are taken up by PC cells via endocytosis, direct membrane fusion, or receptor-mediated uptake. (4-5) The exosomal payload starts a complete reprogramming in the cancer cell’s cytoplasm once it is internalized. Oncogenic signaling pathway activation, metabolic rewiring toward glycolysis, chemoresistance development, immunosuppressive phenotype establishment, and EMT induction for metastasis promotion are all driven by this cargo, a hallmark of PC progression.

CAFs-EXOs are essential mediators that drive the aggressive biology of PC. Their varied molecular payload permits a multi-pronged attack on the TME, controlling critical markers of cancer growth. Furthermore, by secreting EXOs containing regulatory ncRNAs, CAFs contribute to multidrug resistance in PDAC (51). These EXOs transfer specific miRNAs, such as miR-21, miR-146a, miR-106b, and miR-423-5p, and lncRNAs, such as H19, CCAL, and AFAP1-AS1, to cancer cells, where they activate key pro-survival and resistance pathways. The transplanted ncRNAs drive resistance by: I) Activating oncogenic signaling: Upregulating pathways include PI3K/AKT, Signal Transducer and Activator of Transcription 3 (STAT3), and Wnt/β-catenin, which increase cell survival, stemness, and metabolic reprogramming. III) Inhibiting cell death: Suppressing apoptosis by targeting pro-apoptotic proteins, such as APAF1, Bak, and halting ferroptosis by inhibiting critical enzymes, including ACSL4 and ALOX15. (III) Inducing EMT: By targeting EMT suppressors with miRNAs, a metastatic, therapy-resistant phenotype is promoted. IV) Enhancing DNA repair: Facilitating repair of drug-induced DNA damage, lowering apoptotic triggers. V) Modulating drug transporters: Influencing the expression of efflux pumps and other transporters. This EXO-mediated reprogramming helps PDAC cells to survive chemotherapy, such as gemcitabine (GEM) and oxaliplatin, making CAF-EXOs and their ncRNA cargo important therapeutic targets to overcome chemoresistance (51–53) (**Figure 2**).

### 3.1 Driving tumor proliferation, invasion, and metastasis

In cancer cells, essential pathways, such as exosomal SHH and Wnt10b, drive uncontrolled proliferation and EMT, affecting proteins such as Hedgehog and Wnt/β-catenin. Additionally, CAF-EXOs that express specific miRNAs, such as miR-21 and miR-92a-3p, promote metastasis, enhance stemness, and repress tumor suppressor genes. In addition to direct signaling, CAFs modify the ECM by secreting lysyl oxidases and matrix metalloproteinases (MMPs), which degrade the basement membrane and stiffen the tissue. In addition to laying the groundwork for localized metastasis and distant colonization, this mechanism also establishes physical pathways for cancer cell movement and establishes a biomechanically favorable invasion environment (54–56).

Researchers identified the mechanisms by which CAFs release exosomal miR-421 and the consequences for PC progression. EXOs and CAFs were detected and isolated. CAFs-EXOs were administered to PC cells. The levels of miR-421, sirtuin-3 (SIRT3), and hypoxia inducible factors-1 alpha (HIF-1α) were measured by Western blotting and quantitative polymerase chain reaction (qPCR). To assess the cells’ capacity for migration, invasion, and proliferation, researchers used wound-healing assays, Cell Counting Kit-8 assays, and transwell migration assays. The link between miR-421 and SIRT3 was examined using a dual-luciferase assay and RNA immunoprecipitation. H3K9Ac enrichment in the HIF-1α promoter region confirmed by chromatin immunoprecipitation (f). To further investigate the effects of exosomal miR-421 from CAFs on PC, *in vivo* carcinogenesis tests were conducted. The EXOs and CAFs were effectively separated. Increased cell proliferation, migration, and invasion were observed in PC cells treated with CAF-EXO, and these cells also showed elevated miR-421 levels. SIRT3 expression was upregulated upon miR-421 knockdown. Since SIRT3 is a miR-421 target, the detrimental effects of miR-421 knockdown in PC cells were reversed by reducing SIRT3 expression. HIF-1α expression in PC cells was inhibited by knocking down miR-421 in CAF-EXO. In addition, SIRT3 regulated HIF-1α expression by modulating H3K9Ac. The inhibitory effects of SIRT3 overexpression on PC progression were countered by HIF-1α overexpression, and the inhibitory effects of miR-421 silencing on glycolysis were offset. Additionally, *in vivo* carcinogenesis studies demonstrated that miR-421 knockdown reduced CAF-EXO-driven tumor development. By regulating the SIRT3/H3K9Ac/HIF-1α axis, exosomal miR-421 from CAFs promoted PC progression. This investigation illuminated the molecular mechanism of PC (57).

A bad prognosis for PC is often caused by metastasis. Modulating macrophage polarization is one way EXOs control cancer growth. The primary goal of this investigation was to examine the molecular processes and consequences of CAF-released ECM (EXOs) on macrophage polarization in PC. Yang et al. used flow cytometry and qPCR to examine macrophage polarization after treating THP-1 cells or xenografted tumor mice with CAFs-EXOs. They used the Transwell assay and the scratch test to examine metastasis after coculturing THP-1 cells with BXPC-3 cells. Using qPCR, researchers identified exosomal PTGS2, and western blotting enabled assessment of the NOD1 pathway. Researchers’ findings demonstrated that EXOs enhanced motility, invasion, and EMT in PC cells by promoting M2-type polarization and inhibiting M1-type polarization. M2 to M1 macrophage polarization was aided by the reduction of PTGS2 in CAFs, and its expression was upregulated in EXO-treated macrophages. In addition, EXOs polarized cells via PTGS2-mediated promotion of the NOD1 pathway, and NOD1 inhibition undid this polarization. Furthermore, NOD1 was essential for EXOs-mediated M1/M2 polarization *in vivo*. Finally, CAF-EXOs accelerated PC progression by facilitating M2 macrophage polarization, thereby activating the NOD1 pathway and promoting metastasis (58).

Researchers showed that PC cells release EXOs that attract pancreatic stellate cells (PSCs) to distant places, boosting metastasis. Metastatic cancer cells get the protein Lin28B via cancer-derived EXOs, which activate the Lin28B/let-7/HMGA2/PDGFB signaling pathway. Metastatic niche recruitment of PSCs, the progenitors of CAFs, is facilitated by this activation, which in turn increases PDGFB secretion. By demonstrating that primary tumor EXOs can reprogram cancer cells to attract stromal support, this process identifies exosomal Lin28B as a possible therapeutic target to halt the progression of PC to metastasis (59).

In PC, researchers identified a subgroup of CAFs (CD105+ CAFs) whose EXOs promote tumor cell invasion and proliferation. The new AMPK1-360aa protein is encoded by a circular RNA, circAMPK1, which is found inside these EXOs and serves as the primary effector. To prevent AMPK1 degradation, this protein competes with NEDD4, a ubiquitin ligase. As a consequence, AMPK1 becomes more stable, triggering cellular autophagy and promoting the malignant growth of PC cells (52).

Scientists demonstrated that CSCs actively alter the TME by transforming regular fibroblasts into CAFs via EXO secretion. Research has shown that EXOs produced from Piwil2-induced CSCs (Piwil2-iCSC-EXO) may enhance fibroblast proliferation, migration, and invasion by transferring their cargo to fibroblasts. The expression of MMP2/9 and CAF activation markers (α-SMA, vimentin, FAP) increased throughout this metamorphosis. The results show that CSC-stroma communication is crucial in creating a hostile TME, and that CSC-derived EXOs promote tumor growth via reprogramming stromal fibroblasts into a pro-tumorigenic, CAF-like state (60).

Researchers showed that CAF-EXOs are powerful drivers of PC progression through specific molecular pathways, including miR-421/SIRT3/HIF-1α and PTGS2/NOD1. Still, they also highlight significant challenges in translating these findings into clinical practice. The majority of studies use xenograft and reductionist *in vitro* models that don’t fully represent the cellular diversity and dynamic reciprocity observed in a real TME. Additionally, these studies examine only individual exosomal cargos and linear routes, which may not account for the complex, diverse cargo within a single EXO population or the unique network of activities specific to CAF subtypes. The fact that CAF-EXO signals vary spatially, temporally, and patient-specifically highlights a significant information vacuum in this area. Therefore, to create complete, therapeutically relevant exosomal “barcodes,” future research should merge multi-omics investigations of patient-derived EXOs with sophisticated *in vivo* models, such as genetically altered mouse models and patient-derived organoids. To effectively disrupt these protumorigenic communication highways without eliciting the adverse stromal reactions observed in previous broad-stromal targeting trials, the ultimate goal should be to develop smart therapeutics. These could be engineered EXOs for targeted cargo blockade or nanoparticles (NPs) for specific CAF-EXO inhibition.

### 3.2 Orchestrating immune evasion

Tumor immunotherapy has made great strides in recent years. Nevertheless, tumor immunotherapy, especially with anti-PD-1/PD-L1 immune checkpoint drugs, is only successful for a limited fraction of solid cancer patients. There is an immediate need to find a solution to improve the effectiveness of cancer immunotherapy. It is well known that tumor immunotherapy efficacy is heavily influenced by the TME, and recently, there has been significant focus on CAFs within it. A key component of the TME, CAFs interact with cancer cells and immune cells through the secretion of vesicles and cytokines, their involvement in ECM remodeling, and their impact on the immune response. New approaches to identifying targets for combination immunotherapy and estimating immunological effectiveness have emerged from comprehensive research on CAF heterogeneity (61).

Aside from the low tumor mutation load, immunotherapy presents challenges in PC due to the abundance of stromal cells in the microenvironment. Therapies targeting CAFs, either alone or in combination with immunotherapies, are now in development because of CAFs’ vital role in stromal-mediated immunotherapy resistance. At the same time, PC distant receptor cell behavior may be changed by microbiomes and tumor-derived EXOs (TDEs). To better understand PC carcinogenesis and identify companion biomarkers for immunotherapy, it is necessary to understand the roles of CAFs, microbiomes, and TDEs. Biomarkers of PC immunity may be identified through spatial single-cell analyzes of TMEs. Also, artificial intelligence (AI) models will help predict the effectiveness of immunotherapy, as the immune system is highly complex (62).

PDAC TME gives rise to a subpopulation of cells called apCAFs. Because they regulate the immune system and provide structural support, they play an essential role. While apCAFs do produce Major Histocompatibility Complex (MHC)-II molecules, they lack the co-stimulatory signals found in professional antigen-presenting cells. Instead of effectively activating T cells, this “incomplete” antigen presentation renders them functionally inert, a condition known as anergy. In addition, the release of regulatory cytokines, such as TGF-β and IL-10, by apCAFs further contributes to the immunosuppressive environment. High levels of the MHC-II chaperone and cell surface receptor CD74 are characteristic of apCAFs. In the evolution of PDAC and its immune evasion, CD74 plays a pivotal, multifunctional role. It works in three different ways: To begin, CD74 blocks immune surveillance by chaperoning MHC-II, which, in turn, regulates and may decrease the presentation of tumor antigens on apCAFs and cancer cells. Secondly, the activity of cytotoxic T cells and natural killer cells is inhibited by the overexpression of key immunosuppressive mediators, such as prostaglandin E2 and indoleamine 2,3-dioxygenase (IDO), induced by CD74 signaling. Lastly, the activation of tumor cell proliferative and survival cascades, such as the NF-κB and MAPK pathways, is directly driven by the CD74 pathway, which, in turn, fuels tumor development and malignancy (63). Overall, the apCAF/CD74 axis in the PDAC TME represents a powerful immunosuppressive mechanism. The fact that it both provides pro-tumorigenic signals and actively disarms anti-tumor immunity makes it a plausible and consistent target for new immunotherapeutic approaches aimed at breaking down this protective stromal barrier.

CAFs interact with various immune cells to create an immunosuppressive microenvironment in pancreatic tumors. By releasing molecules such as CXCL12, IL-6, and vascular endothelial growth factor, CAFs activate myeloid-derived suppressor cells (MDSCs), which, in turn, limit T cell activity. They promote neutrophil NETosis via amyloid-β and polarize macrophages toward an M2 pro-tumor phenotype via IL-6, M-CSF, and IL-33 signaling. Moreover, CAFs directly inhibit T cell activity by excluding cytotoxic T cells from tumors via ECM remodeling (e.g., collagen deposition) and CXCL12 production, and by encouraging regulatory T cell (Treg) proliferation and T cell anergy/exhaustion in certain subsets (iCAFs, apCAFs) (64). Furthermore, the polarization of macrophages toward a pro-tumor M2 phenotype is one mechanism by which CAF-derived exosomal miR-320a promotes tumor growth. Macrophages receive miR-320a-enriched EXOs from CAFs, which then downregulate PTEN and activate the PI3Kγ pathway, leading to M2 polarization. These M2 macrophages subsequently accelerate PC cell invasion and proliferation. An important mediator of stromal-immune interactions and a possible therapeutic target to disrupt the immunosuppressive milieu in PC is the exosomal miR-320a/PTEN/PI3Kγ axis, which may be blocked by inhibiting miR-320a in CAFs, thereby mitigating this impact (65).

Immunotherapy for PDAC is hindered by this CAF-driven immunosuppression. As a result of stromal exclusion, it probably inhibits chimeric antigen receptor T cell therapy, reduces the efficacy of cancer vaccines by suppressing DC and T cell activity, and limits T cell penetration, which helps explain why checkpoint inhibitors fail. To circumvent this, immunotherapeutic approaches can be used to sensitize PDAC by remodeling the TME and restoring anti-tumor immunity through reprogramming CAFs, depleting specific CAF subsets, such as FAP+ or LRRC15+ myCAFs, or inhibiting CXCL12 (64).

By reprogramming tumor-associated macrophages, this work shows that CAF-EXOs enhance the spread of PC. Macrophage polarization towards an immunosuppressive M2 phenotype is shifted by CAF-EXO, which suppresses the anti-tumor M1 phenotype. The exosomal PTGS2 (prostaglandin-endoperoxide synthase activates NOD1 signaling in macrophages, driving this polarization. As a result, M2 macrophages promote the migration, invasion, and EMT of cancer cells. Significantly, metastatic progression may be hindered by inhibiting PTGS2 or NOD1, which turns this polarization around. According to these results, PTGS2/NOD1 is a potential therapeutic target in PC, and the CAF-EXO-macrophage axis is an essential driver of metastasis (66).

A unique method for immune evasion in PC is promoted by CAF-EVs, according to researchers. This technique is carried out by a specific lncRNA. Once transported to tumor cells, the lncRNA RP11-161H23.5, packaged into CAF-EVs, forms a complex with the mRNA deadenylase subunit CNOT4. This complex cleaves the poly(A) tail of HLA-A mRNA, a molecule essential for antigen presentation, and destroys it. The anti-tumor immune response is hindered by the subsequent downregulation of HLA-A on cancer cells. The researchers have developed a solution: they plan to use modified EVs to deliver siRNA. The goal is to break the immune-evasion axis and maybe get the immune system to recognize itself again. A possible target for improving immunotherapy in PDAC, the RP11-161H23.5/CNOT4/HLA-A pathway is identified as a significant driver of immunosuppression in these results (67).

Despite the undeniable importance of CAFs in PC’s ability to evade the immune system, treatment approaches remain in early stages and face substantial translational challenges, according to a review of the available literature. The main problem is that CAFs are highly heterogeneous, with functionally opposing subtypes (e.g., immunosuppressive apCAFs and possibly immunostimulatory fractions) that are difficult to target with generalized, nonspecific methods. Previous unsuccessful stroma-depleting efforts highlight the counterintuitive acceleration of illness that may result from imprecise disruption of the CAF environment. In addition, the human TME is spatially complex and dynamically reciprocal, and most mechanistic discoveries come from reductionist models that poorly capture these features. Despite the identification of potential targets such as CD74, PTGS2/NOD1, and exosomal lncRNAs, significant knowledge gaps remain regarding the regulation and integration of these pathways across various CAF subpopulations and disease stages.

**Figure 3:**
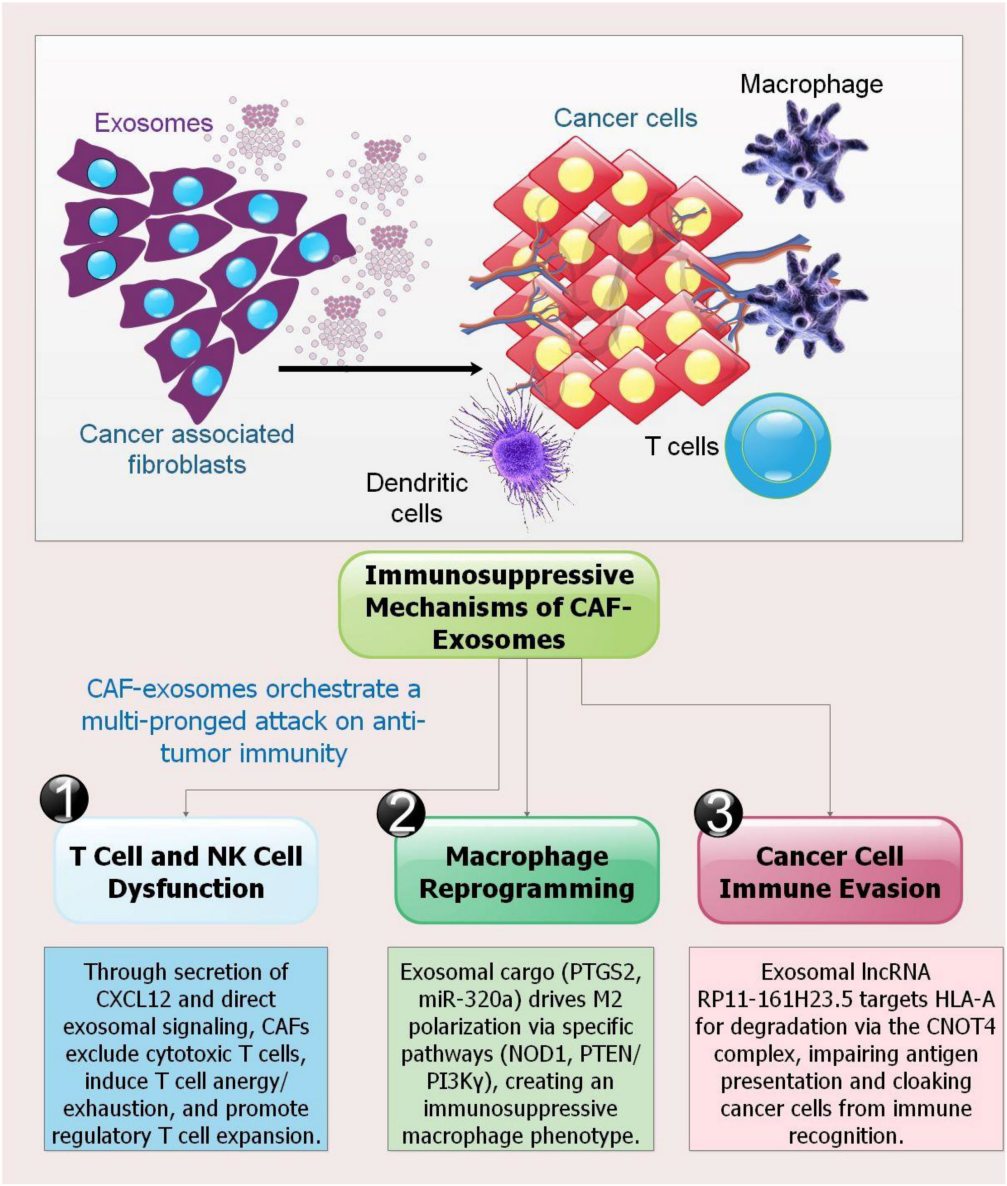
Immune-suppressive mechanisms of CAF-EXOs in the PC TME. CAF-EXOs orchestrate a multi-pronged attack on anti-tumor immunity through three distinct mechanisms: (1) T cell and NK cell dysfunction: Through secretion of CXCL12 and direct exosomal signaling, CAFs exclude cytotoxic T cells from the tumor core, induce T cell anergy and exhaustion, and promote Treg expansion. (2) Macrophage reprogramming: CAF-EXOs transport specific cargos, including PTGS2 and miR-320a, to tumor-associated macrophages. This activates downstream pathways such as NOD1 and PTEN/PI3Kγ, driving polarization toward an immunosuppressive, pro-tumor M2 phenotype. (3) Cancer cell immune evasion: CAF-EXOs deliver the lncRNA RP11-161H23.5 to cancer cells, where it recruits the CNOT4 deadenylase complex to cleave HLA-A mRNA. This results in diminished MHC-I surface expression and impaired antigen presentation to CD8+ T cells, effectively cloaking cancer cells from immune recognition. The combined effects of these processes establish a profoundly immunosuppressive TME that contributes to resistance against immunotherapies (64, 65, 67).

Therefore, precise stromal targeting should be the focus of future efforts. A multi-pronged approach is necessary for this: To begin, we will use state-of-the-art spatial multi-omics on patient samples to identify immunosuppressive niches occupied by CAFs and to construct CAF “barcodes” that are therapeutically important and context-dependent. Secondly, creating innovative treatments, such as modified EXOs or NPs to precisely target specific CAF subsets and deliver medications or siRNA to them, or to disrupt important exosomal cargo produced by CAFs, such as the RP11-161H23.5/CNOT4 system. Thirdly, clinical trials should strategically combine immunotherapies with CAF-targeting drugs, such as CD74 inhibitors and CXCL12 blockers, and use AI-powered models to anticipate individual patient responses. By reprogramming or suppressing aberrant CAF communication, the objective is to transform the immune-hostile TME into a permissive one, enabling effective immunotherapy and ultimately replacing nonspecific stromal ablation (**Figure 3**).

### 3.3 Conferring chemoresistance

In PC, chemotherapy-resistant CAFs actively prolong tumor life by secreting more EXOs in response to GEM. These EXOs promote cancer cell growth and drug resistance by delivering chemoresistance-inducing proteins, including Snail. This protective effect is reversed when EXO release is inhibited with GW4869, demonstrating that CAFs-EXOs are an important mechanism of treatment resistance and a potential therapeutic target to enhance chemotherapy outcomes (68).

It is not well understood how chemotherapy affects CAFs or how CAFs contribute to drug resistance in neighboring cancer cells, despite the long recognition of CAFs’ importance in PDAC. Researchers examined how CAF-secreted EVs (EXOs) influence the phenotypic characteristics of PDAC cells. Richards et al. demonstrated that chemotherapy-exposed CAFs actively control cancer cell survival and proliferation. GEM is the gold-standard chemotherapy drug for PDAC, although CAFs exhibit inherent resistance to it. Additionally, GEM treatment of CAFs greatly enhances EXO release, thereby promoting PDAC epithelial cell proliferation and chemoresistance. At the molecular level, these EXOs enhanced the expression of PTEN-targeting miRNAs and the chemoresistance-inducing protein Snail in recipient epithelial cells. EXO blockade *in vivo* decreased tumor development following GEM therapy, while *in vitro* blockade of CAF EXO release dramatically reduced co-cultured epithelial cell survival, indicating that CAF EXOs play a substantial role in chemotherapeutic drug resistance. The results of this study suggest that EXO inhibitors may be helpful in combination with chemotherapy to combat PDAC chemoresistance (69).

Researchers demonstrated that CAF-EXOs promote a new route of acquired chemoresistance in PC. Even though CAFs lack inherent resistance to GEM, upon GEM treatment, they release EXOs that shield cancer cells from its effects. Translocation of exosomal miR-3173-5p to PDAC cells is the critical mediator of this event. The microRNA-3173-5p inhibits ferroptosis, an iron-dependent cell death mechanism, by targeting and repressing ACSL4 expression in cancer cells. Chemoresistance is conferred by blocking GEM-induced ferroptosis by means of the exosomal miRNA’s inhibition of ACSL4. To combat GEM resistance in PC, this identifies the CAF-EXO miR-3173-5p/ACSL4 axis as an attractive therapeutic target (53). PC patients develop resistance to GEM because of miR-106b, an exosomal CAF-derived miRNA. When CAFs and their EXOs are treated with GEM, miR-106b levels rise. These EXOs deliver miR-106b to cancer cells, where it inhibits TP53INP1, a tumor suppressor gene, allowing cancer cells to survive and fight drugs. A potential therapeutic target to overcome chemotherapy resistance is the exosomal miR-106b/TP53INP1 axis, which may be reversed by inhibiting miR-106b in CAFs (70).

In PDAC, CAFs play a key role in therapeutic resistance by overproducing ECM and altering its composition. Chemotherapeutic agents, such as liposomal doxorubicin, are severely limited in their ability to penetrate the tumor core and deliver cytotoxic T cells into this thick, fibrotic stroma. As a result, immunotherapy and chemotherapy are both rendered useless. Moreover, the ECM serves as a bioactive reservoir, secreting and storing growth factors, such as TGF-β, and providing ligands that directly inhibit immune cell activity. In addition to their barrier roles, CAFs actively promote drug tolerance in cancer cells by secreting EXOs and cytokines such as IL-6. These factors stimulate stemness and EMT, and alter the ECM to create migratory tracks that facilitate metastasis. Essentially, CAFs make PC cells resistant to many drugs by creating a protective, immunosuppressive, and pro-invasive microenvironment around them (71).

New evidence points to CAF-EXOs as a critical, multifaceted chemoresistance mechanism in PC. But there are still significant holes that prevent clinical translation. Snail, miR-3173-5p, and miR-106b are just a few examples of exosomal cargos identified by the field’s reductionist, pathway-specific approach, along with their linear downstream targets. This fails to account for the fact that exosomal payloads are complex and diverse, and that several resistance-promoting elements can be delivered simultaneously. Additionally, research frequently uses xenograft or *in vitro* co-culture models, which poorly simulate the spatial barriers and ever-changing pharmacokinetics of the human pancreatic TME and do not differentiate between the roles of myCAFs and iCAFs in resistance mechanisms.

Integrative and translational approaches should be prioritized in future studies. Top concerns include: One approach is to compare patient samples taken before and after treatment using spatial transcriptomics and proteomics. This will help identify which CAF subpopulations are locally generating resistance-inducing EXOs. 2) creating bioengineered EXO traps or intelligent medication delivery systems that may obstruct several exosomal cargos or pathways all at once, including targeting miRNAs and Snail. 3) Continuing research beyond GW4869 in the search for effective inhibitors of EXO synthesis that are selective and do not interfere with everyday intercellular communication in healthy tissues. 4) Using liquid biopsy monitoring of exosomal cargo as a dynamic indicator of response, design combination clinical trials that rationally link EXO pathway inhibitors with conventional chemotherapy. Researchers want to design precise stromal therapeutics that disrupt the CAF-constructed protective niche, rather than relying on descriptive cataloging of resistance mechanisms.

### 3.4 Metabolic reprogramming

In the nutrient-poor, hypoxic pancreatic TME, CAFs are crucial metabolic partners for cancer cells. Hypoxia promotes cancer cell migration and invasion by rewiring CAF metabolism and inhibiting the tumor suppressor FBXW7. 1) Metabolism of lipids: PDAC cells get the cholesterol and unsaturated fatty acids needed for membrane formation from CAFs. They secrete metabolites like lysophosphatidylcholine (LPC), enter EVs (EXOs), and come into contact with cell membranes (via ANO6) to transfer lipids (72–74). 2) Ketone and carbohydrate metabolism: Lactate is secreted by cancer cells as a result of excessive glycolysis. Through MCT1, CAFs get this lactate, which they then use to power their own TCA cycle and release cytokines that inhibit the immune system, such as IL-6. As a reciprocal mechanism, CAFs reintroduce lactate into the cancer cell energy generation pathway via MCT4. Histone lactylation is one mechanism by which this lactate-rich environment promotes tumor growth (74–76). 3) Metabolism of amino acids: Essential amino acids are supplied by CAFs. To support cancer cell metabolism and prevent ferroptosis, they release alanine (via autophagy) and cysteine (by the transsulfuration pathway). Additionally, they promote chemoresistance through mechanisms such as collagen deposition and activation of branched-chain amino acid metabolism in cancer cells (64, 74, 77). In conclusion, CAFs enhance tumor survival, growth, immune evasion, and therapeutic resistance within the severe TME by undergoing substantial metabolic reprogramming to provide lipids, lactate, and amino acids to PDAC cells.

Researchers uncovered a new mechanism by which CAFs make PC resistant to oxaliplatin. It involves a specific circular RNA, circABCC4. In individuals with PDAC, CircABCC4 is linked to a worse response to chemotherapy and a lower chance of survival. It improves the interaction between the glycolytic enzyme PKM2 and the nuclear transport protein KPNA2, enabling PKM2 to translocate into the nucleus in CAFs. By rewiring CAF metabolism to favor glycolysis, this nuclear PKM2 functions as a transcriptional coactivator. Chemoresistance is promoted by increased DNA damage repair in nearby cancer cells, driven by CAF-produced IL-8. Reversing this resistance *in vivo* was achieved by limiting PKM2 nuclear translocation, which highlights the potential of circABCC4 and the PKM2 nuclear translocation axis as therapeutic targets to overcome chemotherapy resistance (76).

PC cells and CAFs engage in a new metabolic interaction mediated by EXOs, according to the researchers. EXOs contained the lncRNA NNT-AS1, which is abundantly expressed in CAFs and acts as a mediator. Exosomal NNT-AS1 acts as a molecular sponge for miR-889-3p upon binding to PDAC cells, functioning as a competing endogenous RNA (ceRNA). The inhibitory effect of miR-889-3p on its target, HIF-1α, may be alleviated by NNT-AS1. The cancer cells undergo glycolytic reprogramming due to HIF-1α overexpression, which enhances their ability to metabolize glucose, proliferate, and spread. In PC, the NNT-AS1/miR-889-3p/HIF-1α axis is a key player in metabolic remodeling of the TME and offers novel therapeutic options to prevent stromal-tumor communication (78).

This proteomic study described the protein cargo of EVs from normal pancreatic epithelial cells, CAFs, and PC cells. Over 1,400 proteins were identified in each type of extracellular vesicle, with a standard set shared by all groups involved in exocytosis and vesicle transport. Proteins associated with critical tumorigenic processes, such as coagulation, complement activation, and the EMT, were noticeably more abundant in EVs derived from cancer cells and CAFs. These results emphasize the importance of EVs in PC evolution by establishing them as active players in the TME (79) (**Table 1**).

**Table 1:** Multifunctional roles of CAF-Derived EXOs in PC pathogenesis.

Functional outcome	Key exosomal cargo	Target/pathway in recipient cells	Biological effect	References
Tumor Proliferation, Invasion and Metastasis	Proteins: SHH, Wnt10b; miRNAs: miR-21, miR-92a-3p, miR-421	Hedgehog, Wnt/β-catenin pathways; SIRT3/H3K9Ac/HIF-1α axis	Drives uncontrolled proliferation, EMT, stemness, and metastasis. miR-421 enhances glycolysis, migration, and invasion via SIRT3/HIF-1α signaling.	(54–57)
	Protein: Lin28B; circRNA: circAMPK1 (encoding AMPK1-360aa)	let-7/HMGA2/PDGFB pathway; NEDD4/AMPK1/autophagy axis	Lin28B recruits PSCs and CAFs to the pre-metastatic niche; circAMPK1 stabilizes AMPK1, inducing autophagy and promoting malignancy.	(52, 59)
	Protein: PTGS2 (Prostaglandin-endoperoxide synthase 2)	NOD1 pathway in macrophages	Promotes M2 macrophage polarization, which enhances cancer cell motility, invasion, and EMT.	(58, 66)
Immune Evasion	miRNA: miR-320a	PTEN/PI3Kγ pathway in macrophages	Drives M2 macrophage polarization, increasing immunosuppression and tumor growth.	(65)
	lncRNA: RP11-161H23.5	CNOT4/HLA-A axis in cancer cells	Reduces HLA-A (MHC-I) expression on cancer cells, impairing antigen recognition by T lymphocytes.	(67)
	General CAF role (apCAFs)	CD74/MHC-II-mediated T cell anergy; secretion of TGF-β, IL-10, PGE2, IDO	Induces cytotoxic T cell exclusion, M2 macrophage polarization, MDSC recruitment, and T cell anergy/exhaustion.	(61–64)
Chemoresistance	Protein: Snail; PTEN-targeting miRNAs	EMT and survival pathways	Enhances cancer cell survival and chemoresistance in response to GEM. Inhibiting exosome release reverses resistance.	(68, 69)
	miRNA: miR-3173-5p	ACSL4 (ferroptosis regulator)	Confers chemoresistance by suppressing GEM-induced ferroptosis.	(53)
	miRNA: miR-106b	TP53INP1 (tumor suppressor)	Promotes survival and GEM resistance by inhibiting TP53INP1 expression.	(70)
	circRNA: circABCC4	KPNA2/PKM2 nuclear translocation in CAFs	Reprograms CAF metabolism toward glycolysis and increases IL-8 production, enhancing DNA repair and oxaliplatin resistance in cancer cells.	(76)
Metabolic Reprogramming & Support	lncRNA: NNT-AS1	miR-889-3p/HIF-1α axis in cancer cells	Acts as a ceRNA to upregulate HIF-1α, driving glycolytic reprogramming, proliferation, and metastasis.	(78)
	Lipids, metabolites (e.g., LPC), miR-3173-5p	Ferroptosis (ACSL4); membrane synthesis; energy production	Supplies cancer cells with essential lipids, lactate, cysteine, and alanine, supporting survival, proliferation, and therapy resistance in a nutrient-poor TME.	(64, 72–77)
CAF Reprogramming & TME Remodeling	Cargo from Piwil2-iCSC-EXOs (Cancer Stem Cell-derived)	Fibroblast activation pathways	Transforms NFs into activated CAFs expressing α-SMA, vimentin, FAP, and MMPs, reshaping the TME.	(60)
	Proteins promoting EMT, complement, coagulation	Various pathways in recipient cells	EVs from cancer cells and CAFs are enriched with proteins involved in EMT, immune regulation, and coagulation abnormalities.	(79)
General Multidrug Resistance	miR-21, miR-146a, miR-106b, miR-423-5p; lncRNAs (H19, CCAL, AFAP1-AS1)	PI3K/AKT, STAT3, Wnt/β-catenin; apoptosis (APAF1, Bak); ferroptosis (ACSL4, ALOX15); EMT; DNA repair; drug transporters	Increases resistance to oxaliplatin and GEM while promoting cell survival and stemness via epigenetic regulation.	(51–53)

Several significant limitations prevent the practical translation of the current research, which firmly establishes CAFs as master metabolic reprogrammers and EXO-mediated communicators in PC. The full spatiotemporal complexity and the varied subpopulations of CAFs within the dynamic human TME cannot be captured by relying on *in vitro* or simpler *in vivo* models, a fundamental weakness. The majority of research focuses on the coordinated delivery of multiple exosomal cargos (proteins, lipids, various ncRNAs) rather than on individual pathways, such as circABCC4/PKM2, NNT-AS1/miR-889-3p, or specific metabolites. Additionally, at different phases of illness or in response to treatments, little is known about the upstream cues that govern which specific metabolic or pro-tumorigenic program a CAF adopts and bundles into EXOs.

This means that moving forward, studies need to be more integrated, patient-centered, and geographically resolved. As part of this effort, longitudinal patient samples will be analyzed using spatial multi-omics to track the changes in CAF subpopulations and the exosomal markers associated with them as the illness and therapy develop. We urgently need cell-type-specific, innovative, multi-targeted methods that can inhibit PKM2 nuclear translocation, sequester oncogenic lncRNAs, or create tailored EXOs or NPs to disrupt these metabolic communication axes. Transforming CAF from an ally that supports tumors into an antagonist within the TME requires a shift from describing and cataloging pathways to therapeutically altering its metabolic state.

Cancer research has traditionally used the conventional two-dimensional (2D) *in vitro* cell growth technique. Due to the absence of cellular contact (cell-cell and cell-matrix) and communication (cell-cell), this culture cannot replicate the normal TME (80). Loss of heterogeneity, selection bias, clonal evolution, stroma replacement, TME changes, host cell carryover and contaminations, human-to-host cell oncogenic transformation, human and host viral infections, and restrictions on immunologic research are some of the difficulties that must be recognized (81). Although they have limitations, since the murine immune system differs substantially from the human immune system, mouse models allow us to learn about tumor biology in complex, dynamic physiological systems (82). Pancreatic patient-derived xenograft (PDX) models are useful, but they have drawbacks. First, the loss of human stroma in early passages is a characteristic shared by all models, including PDX models. Murine stroma often replaces human stromal tissue in the first generation, and each study must address the significance of the human PDAC tumor-stroma interaction. Second, immunocompromised mice are used to generate PDX tumors. The bulk of stromal tissue and any remaining immune-competent cells in PDX models are derived from mice (83). Therefore, when analyzing the translational implications of recent discoveries, these constraints should be carefully considered.

## 4 Diagnostic potential: CAF-EXOs as liquid biopsy biomarkers

An exciting non-invasive method for diagnosing and assessing the prognosis of PC is liquid biopsies, especially those that examine circulating EXOs. Because EXOs retain the same molecular makeup as their parent cells, they can be used to identify cancer-related mutations, changes in microRNA levels, and alterations in lncRNA levels in blood samples from patients. Crucial information on tumor development, progression, and treatment resistance is carried by the cargo of circulating EXOs produced from both tumors and stroma in PC, particularly those from CAFs. Offering a real-time window into the pancreatic TME, EXO analysis is now positioned as a valuable source of diagnostic, prognostic, and even predictive biomarkers (84–87). Furthermore, rather than freely circulating miRNAs, exosomal miRNAs in saliva and blood have been shown to be more valuable diagnostic and prognostic indicators for PC (88). Independent predictors of recurrence and poor survival post-resection include specific exosomal miRNAs such as miR-17-5p, miR-21, miR-451a, miR-1246, and miR-196a, which are significantly elevated in patients and show better accuracy for early detection. When it comes to managing PC, exosomal miRNA analysis is a game-changer, thanks to its stable, tumor-specific markers that enable early identification, risk classification, and tracking of therapy response (89–93).

Although essential subgroups such as myCAFs, iCAFs, and apCAFs have been identified by single-cell RNA Sequencing (scRNA-seq), the populations that comprise these subtypes differ substantially across cancer types and disease stages. While iCAFs are associated with stemness and chemoresistance, apCAFs have contradictory functions in immunity. Functional subgroups such as mCAFs, vCAFs, and tCAFs are associated with distinct ECM, metabolic, or angiogenic processes. Nevertheless, there is substantial phenotypic overlap and inconsistent terminology, making this categorization difficult and context-dependent. Underscoring the need for combined spatial, molecular, and functional profiling to yield unified CAF classifications relevant to therapy (94).

The mass of pancreatic tumors is made up of 60–70% CAFs. Despite their mesenchymal appearance and lack of non-mesenchymal markers, these cells may originate from a wide range of sources, including PSCs, adipocytes, endothelial cells, mesothelial cells, and MSCs. This variety is the foundation for the considerable variation in CAFs. For instance, in response to tumor signals, activated PSCs may differentiate into myofibroblasts that secrete matrix; apCAFs can inhibit T lymphocytes; and adipose-derived stem cells can differentiate into various subtypes (myCAFs or iCAFs). Due to their diverse origins and functions, CAFs constitute an essential and highly heterogeneous component of the pancreatic TME (88).

PC indicators of tumor-stroma crosstalk and disease progression include exosomal miRNAs derived from CAFs. Upon chemotherapy (e.g., GEM) and subsequent transfer to cancer cells, certain miRNAs are increased in CAF EXOs. These miRNAs confer chemoresistance by targeting genes, including TP53INP1. To further illustrate the non-invasive diagnostic and prognostic use of exosomal miRNAs, consider the following: increased serum levels of miR-21 and miR-17-5p are associated with metastasis, advanced stage, and poor survival. Furthermore, PDAC-derived exosomal miRNAs, including miR-1246, miR-30c, and miR-181a, can potentially prime the liver pre-metastatic niche by activating hepatic stellate cells, highlighting their role in metastasis. As a result, CAF-exosomal miRNAs serve a twofold purpose: first, as indicators for early identification, patient stratification, and progression monitoring of PC; and second, as therapeutic targets to combat resistance (95–99).

Scientists have uncovered a process by which PC cells alter the tumor’s surrounding environment. Adjacent NFs take up microvesicles released by cancer cells that have high concentrations of miR-155. Once inside, exosomal miR-155 targets and suppresses the tumor suppressor gene TP53INP1, promoting the conversion of these NFs into CAFs. This indicates that EVs originating from tumors play a crucial role in influencing the stromal environment by transporting targeted miRNAs. Therefore, a potential treatment approach to decrease CAF activation and disrupt tumor-stroma crosstalk in PC is to target this circulating miR-155/TP53INP1 axis (100).

EVs loaded with Annexin A1 (ANXA1) provide a new channel for PC cells to interact with and alter the tumor stroma, as shown by researchers. Tumor cells release EVs, which activate fibroblast and endothelial cell formyl peptide receptors (FPRs). Fibroblasts undergo cytoskeletal remodeling and a phenotype linked with cancer known as activated migratory myofibroblasts as a result of this ANXA1/EV-FPR signaling. It also promotes angiogenesis by increasing endothelial cell mobility. This means the ANXA1/EVs-FPR axis is an important paracrine mechanism that promotes tumor growth, angiogenesis, and stromal activation. The results suggest that ANXA1, whether in soluble or circulating EV form, may be a useful diagnostic or prognostic biomarker for PC and a therapeutic target for blocking tumor-stroma communication (101). A new protein complex, Annexin A6 (ANXA6)/LRP1/TSP1, is identified as a critical factor in PC aggressiveness, acting via EVs produced by CAFs. The complex is packaged into ANXA6+ EVs and is exclusively expressed in CAFs under tumor-like conditions. PC cells absorb these EVs, which enhance their survival, migration, and metastasis. Whereas infusing ANXA6+ EVs enhances tumor development, depleting ANXA6 in CAFs hinders it. According to clinical data, ANXA6+ EVs are detectable in the serum of patients with PDAC and are associated with tumor grade. This suggests that they may serve as useful diagnostic biomarkers and as potential therapeutic targets for disrupting this important communication pathway between the tumor and the stroma (102).

**Figure 4:**
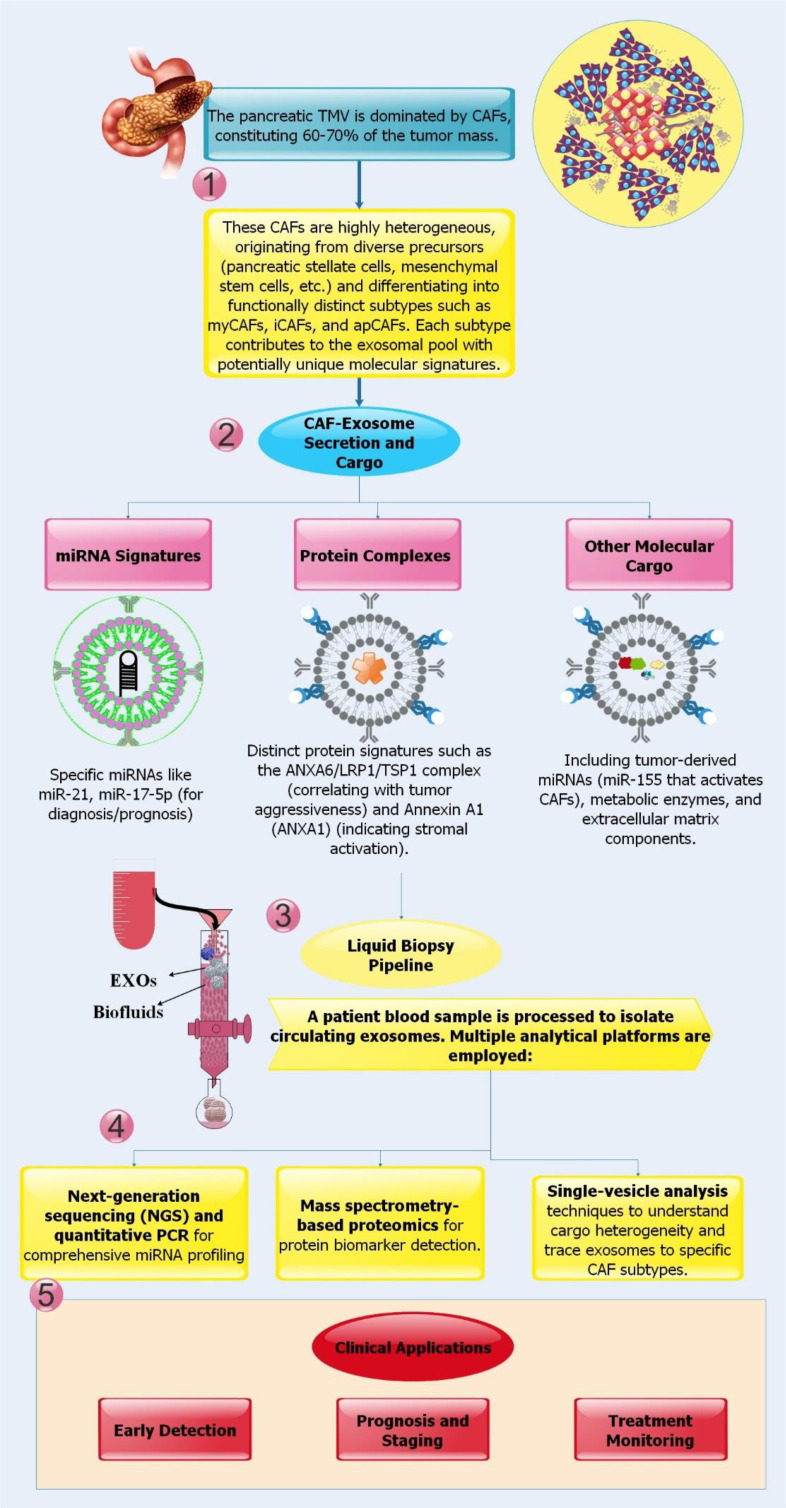
(1-4) CAF-EXOs are a powerful liquid biopsy diagnostic for PC because they carry many molecular markers of active tumor-stroma crosstalk in patient blood, including particular miRNAs (miR-21, miR-106b) and protein complexes (ANXA6/LRP1/TSP1). (5) EXOs extracted from blood samples and examined using methods like mass spectrometry and next-generation sequencing provide a non-invasive insight into the TME, despite continuous difficulties with standardization and clinical validation. Early discovery, precise staging and prognosis, and real-time tracking of therapeutic response and chemoresistance development are all made possible by this.

Researchers demonstrated critical tumor-stroma communication pathways and have significant potential for non-invasive biomarkers in CAF and EXO biology in PC. Still, many questions remain unanswered. For example, current EXO research often treats “CAF-EXOs” as a single entity, potentially leading to the conflation of competing biological signals rather than to the resolution of the remarkable heterogeneity and functional duality of CAFs. The absence of standardized procedures for isolating EXOs, the challenge of positively associating EXOs with specific CAF subtypes in patient blood, and the need for extensive clinical validation of potential biomarkers are all examples of technical constraints. Prospective trials should be launched to validate lead biomarkers, such as ANXA6+ EVs, for early detection and monitoring, and to exploit exosomal communication pathways as novel therapeutic targets. Recent advances in single-cell and spatial multi-omics techniques have uncovered unique functional subpopulations within the TME and offered greater insights into CAF heterogeneity. Future directions should also include integrating single-vesicle and spatial multi-omics to link exosome cargo to specific CAF subpopulations and to develop CAF-specific EXO capture technologies (**Figure 4**).

CAFs have a crucial role in modulating immunotherapy-induced primary resistance. EXOs are a relatively new area of study, as recent data suggest that CAFs interact with tumor and immune cells via these vesicles. But there is still much to learn about this system. Size-exclusion chromatography, binding and enrichment kits, differential ultracentrifugation, and ultrafiltration are methods used in exosome research to separate exosomes from bodily fluids. RNA/miRNA, protein/mass spectrometry, and DNA analysis are performed on the separated EXOs. Despite being well-established and well-documented in the literature, these methods primarily provide descriptive datasets and hypotheses. On the other hand, a few experimental methods using monocultures, co-cultures, or organoids enable the repeatable synthesis of EXOs and EXO-free fractions, thereby enabling biomechanistic discoveries and rescue studies. Determining cell-specific effects requires separating EXOs generated by various cellular sources, such as tumor cells and CAFs, which is a more difficult method (43). The high variability of CAFs in tumors is primarily responsible for the lack of clarity about their biological activities in PC, which exhibit both pro- and antitumorigenic qualities, according to prior research. The ambiguity surrounding the biological role of CAFs in tumors is further clarified by the distinct interactions between CAFs and tumor cells. To successfully combat cancer, it is crucial to define the diversity of CAFs and their interactions (52). When combined, these results point to several difficulties in clinical translation. The consistency of exosome-based findings may be affected by heterogeneity in CAF populations, and inter-study variability may arise from inconsistent isolation techniques. Furthermore, clinical validation of the proposed biomarkers is required, as most of the data are currently derived from experimental models.

### 4.1 Impact of CAF-EXO heterogeneity on therapy resistance and biomarker development

According to experimental results, LINC01711 is strongly expressed in CAF-EXOs, which upregulate TXN via the miR-4510/NELFE axis. This promotes the glycolytic pathway, thereby increasing the propensity of BRCA cells to proliferate, migrate, and invade (103).

By connecting stromal adaptation to hypoxia with malignant reprogramming, Lu et al.’s work identified hypoxia-conditioned CAF-derived exosomal circSTAT3 as a crucial modulator of BC aggressiveness. CAF density and histopathological grade were directly correlated in clinical assessments, highlighting their significance in advanced illness. By facilitating stromal-tumor interactions, these EXOs confer chemoresistance and stem-like plasticity to cancer cells (46).

In Zhao’s research, EXOs produced by CAFs enhanced 5-FU resistance in colon cancer cells and tumor xenograft development. MiR-223-3p was markedly elevated in colon cancer patients’ blood or tissue samples, as well as EXOs generated from CAFs. Additionally, NF2 was shown to be a downstream target of miR-223-3p. In colon cancer cells, the effects of miR-223-3p exosomal translocation were partly reversed by restored NF2 expression. Furthermore, by focusing on NF2, exosomal miR-223–3 controlled the Hippo pathway in colon cancer cells (104).

For laryngeal squamous-cell carcinoma (LSCC), miRNAs have been suggested as minimally invasive biomarkers. The areas under the ROC curves for two serum-based case-control studies (111 LSCC, 80 controls) were 0.876 (miR-21 + HOTAIR) and 0.797 (miR-941), with corresponding sensitivities of 94% and 82%, respectively. Seven mechanistic papers showed that vesicular cargos, including miR-1246, circPVT1, and LINC02191, drive STAT3-dependent M2 polarization, NOTCH1-mediated stemness, Rap1b-VEGFR2 angiogenesis, and glycolytic re-programming, producing 1.6–2.6-fold increases in invasion, tube formation, or xenograft growth (105). When combined, these results demonstrate the significant variability in CAF-EXOs and their context-dependent impacts on tumor development. This heterogeneity makes it more difficult to establish reliable exosome-based biomarkers and may help explain some of the variations in therapy response.

## 5 Therapeutic strategies: targeting the CAF-EXO axis

Thanks to their biocompatibility, cargo-protecting capabilities, and rapid cellular absorption, EXOs show great promise as natural delivery vehicles for cancer treatment. Therapeutic proteins, genes, medications, or substances that modulate the immune system may be engineered into them. An example of this is the ability of EXOs containing SIRPα variants to inhibit tumor cell CD47 “don’t eat me” signals, thereby improving macrophage phagocytosis and encouraging T-cell infiltration. Hybrid nanovesicles or pH-responsive EXO-antibody conjugates are advanced designs that further enhance targeted delivery and effectiveness in the TME. Their translational potential has been demonstrated in early clinical studies, such as one that used DC-derived EXOs as a tumor vaccine in non-small cell lung cancer. To fully realize their therapeutic potential in cancer therapy, however, obstacles to standardization, scalable manufacturing, and targeted distribution must be overcome (9, 106, 107). Through exosomal transfer and paracrine signaling (via GM-CSF and IL-6), CAFs activate pathways such as JAK/STAT, mTOR, SHH, and NF-κB, and engage with tumor cells, playing a crucial role in creating the thick, desmoplastic stroma. Autophagy is another metabolic adaptation that helps tumors survive in nutrient-poor environments. CAFs enhance tumor growth, invasion, metastasis, and resistance to chemotherapy. As a result, restricting CAF secretory capabilities or inhibiting pathways such as SHH and JAK2 has become an active area of study in stromal targeting, with the potential to enhance drug delivery and overcome resistance (108).

Given the risks associated with wide stromal ablation, a new approach to treating PC is emerging that targets CAFs. This approach involves precisely modulating certain pro-tumorigenic activities. To rebuild the ECM, decrease chemoresistance, and ameliorate immunosuppression, this process uses targeted inhibition of key pathways triggered by CAF (TGF-β, Focal Adhesion Kinase (FAK), and JAK/STAT) and stromal components (fibronectin and LOX). To put tumor-promoting CAFs into a dormant state, new methods are looking to reprogram them instead of depleting them. These methods use microRNA regulators and markers, such as Meflin. To resensitize PCs to immunotherapy and chemotherapy, the ultimate objective is to interrupt harmful stromal-tumor communication while preserving potential tumor-restraining capabilities (109).

### 5.1 Inhibition of exosome biogenesis and release

Through the delivery of certain miRNAs—miR-21, miR-181a, miR-221, miR-222, and miR-92a—that inhibit the tumor suppressor PTEN, particularly after GEM therapy, CAF-derived EXOs induce chemoresistance in PC. A potential therapeutic approach to overcome chemotherapy resistance is EXO blockade, as demonstrated by the restoration of PTEN expression *in vivo* upon inhibition of EXO release with GW4869 (110).

Researchers identified a new source of tailored medication delivery in PC: the patient’s own non-cancerous fibroblasts. The anti-cancer and anti-desmoplasia medication ormeloxifene (ORM) was effectively loaded into EXOs generated from NFs (NAF-EXs) that are located near tumors. These modified NAF-EXs demonstrated a natural tropism for PCs, enabling them to successfully deliver ORM to the tumor site. By controlling calcium influx, ORM stopped NFs from becoming cancer-promoting CAFs. In a mouse model, NAF-EXs-ORM effectively inhibited tumor development by blocking oncogenic pathways related to desmoplasia and EMT. To improve the effectiveness of PC treatment, our method demonstrates the viability of using autologous EXOs as a biocompatible, tumor-homing delivery mechanism (111).

Using EXOs produced by pancreatic CAFs (pCAFs) and MSCs modified to express the suicide gene yCD::UPRT, researchers demonstrated a new “Trojan horse” treatment approach. By converting the non-toxic prodrug 5-fluorocytosine (5-FC) into the cytotoxic chemotherapeutic agent 5-fluorouracil (5-FU) and its metabolites, these modified EXOs induce targeted tumor cell death when ingested by PC cells. EXOs derived from both cell types showed a dose-dependent inhibition of cancer development in a simulated desmoplastic tumor model. One potential targeted treatment for PDAC is this method, which uses EXOs produced from pCAF and MSC that naturally home to tumors to deliver a powerful, locally tailored chemotherapy directly into the TME (112).

Although it is a novel approach, there are substantial biological and translational challenges associated with blocking EXO synthesis or reusing EXOs as therapeutic carriers. While GW4869 and other pharmacologic inhibitors restore chemosensitivity, broad-spectrum EXO-blocking approaches risk interfering with vital intercellular communication in healthy tissues and causing off-target damage. Engineering autologous EXOs, such as NAF-EXs or suicide-gene EXOs, provides an alternative, more targeted approach by leveraging natural homing to deliver payloads directly to the tumor. On the other hand, there are concerns about the reliability and effectiveness of tailored cargo, and these technologies require scalable, repeatable manufacture of EXOs suitable for clinical use. Furthermore, the root cause of abnormal CAF activation is not addressed by these methods; instead, they focus on the symptom, the pathogenic exosomal cargo. Identifying routes of EXO biogenesis specific to CAF subtypes will enable more targeted interventions, and the reliability and affordability of tailored EXO treatments will determine their potential for broad clinical use.

### 5.2 Blockade of recipient cell uptake

In PC, CAFs pose a significant obstacle to the penetration and effectiveness of traditional chemotherapy; however, nanomedicine offers a strategic approach to circumvent this challenge. These platforms surpass the unreliable enhanced permeability and retention (EPR) effect through NPs designed with exact control over size, surface chemistry, and targeting ligands. This allows them to evade biological obstacles, protect therapeutic cargo, extend circulation, and actively target CAF-specific surface receptors. Metal NPs for integrated imaging and photothermal therapy; polymeric NPs for flexible, multifunctional co-delivery; and cell-membrane-based NPs that mimic real cells to evade immune clearance are important nanomaterial platforms. A more reasonable strategy to overcome the stromal barrier, increase intratumoral drug accumulation, and improve treatment outcomes in desmoplastic cancers such as PDAC is to use active, CAF-targeted nanodelivery devices (113).

Researchers developed an NP system, BMN/GEM@PE, that simultaneously modulates the PC immune microenvironment and the tumor stroma. Coated with GEM, the NPs consist of bovine serum albumin, manganese dioxide, and niclosamide within. This designed platform effectively reduces the frequency of two CAF subtypes, myCAFs and apCAFs, by suppressing STAT3 signaling. Drug penetration and CD8+ T cell infiltration are both improved by decreased myCAFs, which, in turn, reduce ECM density. At the same time, fewer immunosuppressive Tregs are present due to the decline in apCAFs. As a result, the tumor goes from an immunologically “cold” to a “hot” state, thanks to this dual-action NP, which significantly slows down the evolution of the original tumor and the spread of cancer to the liver. In PDAC, researchers’ approach demonstrates that targeted manipulation of specific CAF subtypes can alter the TME, thereby improving the efficacy of combined immunochemotherapy (114).

To circumvent the immunosuppressive barrier in PC, researchers devised a synergistic nano-delivery approach. To reprogram and deactivate CAFs, the researchers developed a nanochaperone (nChap) platform that simultaneously delivers two key agents: the vitamin D receptor ligand calcipotriol (Cal) and the chemokine CXCL9, which actively attract CD8+ cytotoxic T lymphocytes (CTLs). To relax the thick ECM, Cal reverses CAF activation; meanwhile, CXCL9 establishes a chemotactic gradient that draws CTLs into the tumor. By combining Cal@nChap-CXCL9, the therapeutic effectiveness against advanced PCs is much increased, as is the penetration of immunotherapy (anti-PD-1) and chemotherapy (GEM). PCs that are immunologically “cold” may become “hot” with this method, opening the door to successful combination chemo-immunotherapy (115).

There is a lack of understanding of how nanomedicine strategies for stromal reprogramming interact with particular CAF subpopulations in human tumors, the manufacturing complexity of multi-functional platforms, and the oversimplification of preclinical models, all of which impede their clinical translation. Integrating real-time monitoring capabilities, creating more modular and scalable nanotechnologies proven in patient-derived models, and rationally combining them with current medicines based on patient-specific stromal biomarkers are crucial for future success.

### 5.3 Neutralization of oncogenic cargo

EXOs containing the oncogenic microRNA-21 (miR-21) are released by PSCs, which are important CAFs in PDAC. Clinical data analysis reveals that elevated miR-21 levels are associated with worse patient outcomes and suggest that it may target the Ras/ERK signaling pathway. Research conducted in a controlled laboratory setting shows that miR-21 levels are elevated when PDAC cells absorb EXOs produced by PSCs. This absorption increases MMP activity, triggers the EMT, and promotes cancer cell migration. Important signaling pathways are activated, leading to the process, which includes increased phosphorylation of ERK1/2 and Akt. According to these results, exosomal miR-21 derived from PSCs is an important factor in PDAC and a potential therapeutic target (116).

In a work, researchers identified a new mechanism by which CAFs use exosomal signaling to promote pancreatic tumor formation. PC cells absorb the hormone leptin from EXOs released by CAFs. Within these cells, the oncoprotein ABL2 is upregulated by exosomal leptin, which inhibits the tumor-suppressing microRNA miR-224-3p. Cancer cell migration, invasion, and proliferation are driven by the ensuing increase in ABL2. Notably, this tumor-promoting impact is reduced by suppressing Leptin or overexpressing miR-224-3p, but rescued by restoring ABL2 expression. As a possible therapeutic target, our data highlight the CAF exosomal Leptin/miR-224-3p/ABL2 axis as a critical driver of PDAC development (117).

Scientists have uncovered a novel pathway by which CAF-EXOs promote PC development. One critical effector is miR-125b-5p, a microRNA transported from CAFs to cancer cells via EXOs and highly expressed in pancreatic tumors. Upon entry into cancer cells, exosomal miR-125b-5p stimulates EMT, metastasis, and cell proliferation. At the molecular level, it binds to the 3’-untranslated region of the tumor suppressor gene adenomatous polyposis coli (APC), thereby inhibiting its expression. This suppression of the APC accelerates tumor development and spread. The results show that exosomal miR-125b-5p is an essential factor in CAF-driven cancers and a potential target for treating PC by preventing stromal-tumor communication (118).

By changing cancer cell metabolism, researchers showed that exosomal miR-421 from CAFs causes PC growth. When CAF EXOs reach PC cells, miR-421 binds to and inhibits SIRT3, a mitochondrial deacetylase. The transcription of the crucial metabolic regulator HIF-1α is enhanced as a result of the downregulation of SIRT3, which causes an increase in the acetylation of histone H3K9. This leads to increased HIF-1α levels, which, in turn, improve cancer cell glycolysis, proliferation, migration, and invasion. Reducing miR-421 expression in CAF EXOs reduced tumor development in living organisms. As a result, the exosomal miR-421/SIRT3/H3K9Ac/HIF-1α axis is recognized as a possible therapeutic target and a crucial route by which CAFs metabolically alter the TME (119).

Researchers have unveiled a new approach to treating PC: using modified EVs to alter the TME. The anti-fibrotic medication pirfenidone (PFD) and the microRNA miR-138-5p were co-loaded onto integrin α5-targeting EVs (IEVs) created by researchers using bone marrow MSCs. By altering their surface, CAFs might be precisely targeted. When miR-138-5p was delivered, it hindered two important pathways that promote fibrosis: one was the FERMT2-TGFBR1 complex, which it inhibited to decrease TGF-β signaling, and the other was the FERMT2-PYCR1 complex, which it disrupted to limit collagen production. When administered in conjunction with PFD, this method altered CAFs in a synergistic manner, resulting in less fibrosis, reduced tumor stiffness, improved GEM perfusion, and reduced hypoxia. This CAF-targeted therapy showed great promise in preclinical models for reshaping the immunosuppressive stroma and considerably increasing chemotherapy sensitivity, suggesting a potential approach to combat treatment resistance in PC (120).

Investigators demonstrated that the aggressiveness of PC is driven by microRNA-mediated interaction between cancer cells and stromal cells. In particular, miR-21 and miR-221 expression is much greater in CAFs and PSCs than in normal PSCs and cancer cells in general. The clonogenicity and stem-like features of PC cells were improved by conditioned media from PSCs/CAFs enriched with these miRNAs. At the functional level, blocking miR-21 in PSCs decreased their migration and invasion, while blocking miR-221 in CAFs inhibited invasion and NF-κB and K-Ras signaling. The results show that miR-21 and miR-221, which originate in the stroma, play an important role in the malignant PC phenotype by mediating the communication between tumors and stroma. One potential precision medicine approach to halt tumor growth and enhance treatment results is to target these miRNAs in the stromal compartment (121).

Researchers have uncovered a new mechanism by which EVs secreted by CAFs enhance PC growth by releasing the oncogenic miR-331-3p. PC cells get miR-331-3p, which is abundant in CAF-EVs. MicroRNA-331–3p binds specifically to cancer cells and inhibits expression of the tumor suppressor gene Scavenger Receptor Class A Member 5 (SCARA5). Stimulation of the FAK signaling pathway—a critical driver of proliferation, migration, and invasion—is induced by SCARA5 downregulation. So, miR-331-3p is delivered to cancer cells *in vivo* and *in vitro* by CAF-EV, thereby making cancer cells more malignant. To prevent PC growth and disrupt stromal-tumor communication, this CAF-EV miR-331-3p/SCARA5/FAK axis is a potential therapeutic target (122).

Reprogramming pCAFs using modified EVs was the goal of this study’s precision therapeutic approach. The creation of ITGA5-EVs-148a was achieved by researchers by altering EVs generated from bone marrow MSCs (BMSC) with a peptide that targets integrin α5 (ITGA5) and then loading them with miR-148a-3p, a miRNA that is downregulated in CAFs. Improved targeting and CAF uptake were observed with these modified EVs. The miR-148a-3p inhibitor inhibited the TGF-β/SMAD pathway by targeting ITGA5, thereby decreasing CAF proliferation and migration. By rendering CAFs inactive and reversing their tumor-promoting actions, ITGA5-EVs-148a successfully suppressed tumor development in both 3D models and xenografts. Reprogramming the tumor stroma as a therapeutic target for PC is the focus of this method (123).

Research has shown that certain miRNAs in CAF-EXOs, such as miR-21, miR-125b-5p, miR-421, and miR-331-3p, are potent oncogenic drivers that promote PC development, chemotherapy resistance, and metabolic reprogramming. One major drawback, nevertheless, is the overemphasis on the specific miRNA-target axis in *in vitro* or simplified *in vivo* models. This method fails to account for the complex and diverse composition of exosomal cargoes, which may include proteins (such as leptin), miRNAs, and other compounds, potentially leading to a synergistic pro-tumorigenic signal. In addition, studies do not always connect the dots between these molecular pathways and the functional diversity across CAF subtypes (e.g., myCAFs vs. iCAFs); it is not always evident which subpopulations create which harmful EXOs. Though encouraging, there remains a significant gap in translation regarding scalable production, immunogenicity, and accurate *in vivo* targeting specificity in people, despite modified EVs having been successfully used in preclinical studies to deliver targeted miRNAs. The discipline needs to go forward with patient-centered, integrated techniques. This entails creating multi-target therapies, such as tailored NPs that may neutralize broad oncogenic targets, and using spatial multi-omics to map patient-specific EXO signals by CAF subtype. In the end, we want to use these findings to develop precise stromal reprogramming that improves clinical outcomes by combining chemo- and immunotherapy with EXO-targeting techniques.

## 6 Navigating the storm: challenges in targeting the stromal exosome network

The investigators uncovered a new paracrine pathway induced by mutant KRAS that confers PC chemoresistance. CAFs secrete the oncogenic KRASG12D protein via EVs from tumor cells. Upon entering CAFs, the KRASG12D protein increases their capacity to migrate, proliferate, and, most importantly, induce GEM resistance in cancer cells in both laboratory and *in vivo* settings. This shows that, at a non-cell-autonomous level, KRAS mutations may alter the TME and confer therapeutic resistance. This chemoresistance was successfully countered by the KRASG12D-specific inhibitor, MRTX1133. The results shed light on the poor outcome of patients with KRAS mutations after chemotherapy and support the idea that targeting stromal KRASG12D, which can be delivered by tumor EVs, might be a viable approach to make PCs more responsive to treatment (124).

Results from preclinical and clinical studies targeting the IL-1/JAK/STAT3 pathway in pCAFs have been mixed, particularly regarding the inflammatory iCAF subtype. In mouse models, inhibiting this pathway with IL-1 receptor antagonists (such as Anakinra), IL-1β-neutralizing antibodies, or JAK/STAT3 inhibitors improves responses to immunotherapy and chemotherapy, decreases tumor-promoting inflammation, increases CD8+ T cell infiltration, and decreases fibrosis. But translating it into clinical practice has been tough. Ruxolitinib, a JAK inhibitor, was only effective in a small percentage of patients with high inflammation (high C-reactive protein) in a phase II trial. Subsequent phase III trials did not improve overall survival, suggesting that feedback loops and compensatory bypass mechanisms limit the effectiveness of single-pathway inhibition. Clinical investigations into blocking the IL-1 pathway are ongoing. Still, combination therapy likely targeting redundancy in signaling pathways and the intricate signaling networks responsible for CAF activation in PDAC will be necessary for future strategies (125–127).

As the dominant cellular component of the PDAC stroma, CAFs orchestrate extensive and biologically diverse crosstalk with PC cells and immune cells, contributing to a unique PDAC TME that promotes cancer proliferation, metastasis, and resistance to both chemo- and immunotherapies. This has led to the discovery of CAFs and the processes they mediate, which are now considered potential therapeutic targets for PDAC. Concerns about “what to deliver” and “how to deliver” have arisen during the development of CAF-targeted drug delivery systems to selectively inhibit tumor-supporting CAFs without compromising tumor-restricting CAFs, due to several clinical setbacks and the growing understanding of the PDAC stroma, which has exposed the heterogeneity and complex biological functions of CAFs (128). In PC, more complicated approaches to reprogram CAFs have replaced direct stromal depletion, which may accelerate tumor growth, as this review shows. Some essential methods include making CAFs dormant or altering their behavior to reduce tumor growth, and using substances such as bioactive lipids (e.g., Lipoxin A4), vitamin D analogs (e.g., Cal, all-trans retinoic acid (ATRA)), Minnelide (which blocks TGF-β), curcumin, and ROCK inhibitors (e.g., Fasudil). These therapies enhance medication delivery, regulate the immunological milieu, and reduce myofibroblast activation and ECM deposition. Clinical results have been inconsistent when targeting individual ECM components, such as hyaluronan with PEGPH20, or upstream signaling pathways, such as FAK, FGFR, and JAK/STAT. In pancreatic ductal cancer, available data suggest that AF reprogramming and targeted pathway inhibition may be more effective than broad stromal ablation in improving chemotherapeutic efficacy and patient survival (129).

A key player in the paracrine communication that drives tumor development and immunosuppression in PC cells with a KRAS mutation and CAFs is the CXCR2/CXCL chemokine axis. The oncogenic KRAS gene increases CXCR2 expression in tumor cells and triggers CXCR2 ligand secretion by CAFs (CXCL1, CXCL5, CXCL7). The activation of CXCR2/NF-κB signaling in CAFs is a two-way street: it promotes a secretome that is both pro-inflammatory and tumor-supportive, and it attracts MDSCs, which inhibit the immune system. In preclinical models, inhibiting CXCR2 has been shown to reduce tumor development and increase survival. In a similar vein, the CXCL12/CXCR4/CXCR7 axis plays a pivotal role in stromal-mediated immunological exclusion: CXCL12, produced by CAFs, attracts Tregs and excludes cytotoxic CD8+ T cells, while CXCR7, its receptor, promotes the invasion and metastasis of cancer cells. Interestingly, some stromal chemokine signaling activities may inhibit rather than promote cancer, and paradoxically, total genetic ablation of these pathways (Cxcr2 or Cxcr4 deletion) may occasionally accelerate carcinogenesis. Instead of widespread stromal ablation, targeted stromal modulation is necessary, which may be achieved by inhibiting CXCR2 or CXCR4 and combining immunotherapy (130).

Rather than viewing the stroma as a monolithic, protumorigenic “foe,” this section examines the multifaceted role of CAFs in PC. Contrary to popular belief, CAFs do not always support tumor development. This becomes clear when considering the clinical and preclinical failures of broad stromal elimination therapy. According to recent research, some subpopulations of CAFs, including αSMA-high myCAFs and CD271+ PSCs, can play tumor-restraining roles, and their loss can lead to more aggressive, less differentiated tumors. On top of that, tumor stage, stromal makeup (“normal” vs. “activated” stroma), and environmental stresses all affect CAF function, which is dynamic and context-dependent. Examples of factors with biphasic effects include periostin. It is important to note that the tumor stroma is a complex mixture of forces, some of which promote cancer and others that inhibit it. As a result, current treatment approaches rely on non-specific stromal ablation, which is counterproductive. Instead, researchers need to identify and manipulate specific subpopulations of CAF based on their functional status within the evolving TME (131).

This section discusses new approaches to treating PC that focus on regulating the tumor stroma instead of removing it entirely. Medications such as Minnelide and ATRA may retrain active CAFs into a dormant state, while NPs such as CellaxTM-DTX can specifically target and destroy certain CAF populations. Broad stromal depletion has been unsuccessful in the past (e.g., with SHH inhibitors), underscoring the dangers of removing stromal components that may limit tumor growth and cause side effects, such as enhanced angiogenesis. So, stromal remodeling has become the next hot topic. This involves normalizing the milieu, improving medication delivery, and perhaps enhancing immunotherapy with drugs like vitamin D receptor agonists or JAK/STAT inhibitors. Personalized combinations of stromal-modifying drugs, chemotherapy, and immune checkpoint inhibitors will likely be the key to future success in treating inflammatory and stromal disorders (126, 131–134) (**Table 2**).

**Table 2:** Therapeutic strategies targeting the CAF-EXO axis in PC.

Therapeutic strategy	Approach/agent	Type of study	Mechanism of action	Effect/outcome	Ref
Inhibition of EXO Biogenesis and Release	GW4869 (EXO release inhibitor)	Review	Silences CAFs and their ability to produce EXOs.	Reverses GEM resistance and restores chemosensitivity in cancer cells via increasing PTEN expression.	(110)
Repurposing EXOs as Delivery Vehicles	NAF-EXs-ORM (EXOs from Normal Adjacent Fibroblasts loaded with ORM)	Preclinical (*In vivo*)	Uses tumor-tropic autologous EXOs to transport anti-fibrotic and anti-cancer medication. A standard fibroblast→CAF conversion is prevented.	*In vivo*, it inhibits tumor development, EMT, and desmoplasia.	(111)
Repurposing EXOs as Delivery Vehicles	pCAF-/MSC-EXOs engineered with yCD::UPRT (suicide gene)	Preclinical (*In vitro*/3D Model)	Transforming the prodrug 5-FC into the cytotoxic 5-FU within tumor cells is achieved via engineered EXOs.	Kills tumor cells specifically in a desmoplastic model.	(112)
Blockade of Recipient Uptake/Nanomedicine	BMN/GEM@PE NPs (EXO-coated NPs with BSA-MnO_2_-Niclosamide core + GEM)	Preclinical (*In vivo*)	Inhibits STAT3 in CAFs, which lowers myCAFs and apCAFs.	Inhibits primary tumors and metastases, decreases ECM density, promotes CD8+ T cell infiltration, and suppresses Tregs.	(114)
Blockade of Recipient Uptake/Nanomedicine	Cal@nChap-CXCL9 nChap (Co-delivers Cal and CXCL9)	Preclinical (*In vivo*)	Attracts CD8+ T cells (via CXCL9) and reprograms/deactivates CAFs (via Vitamin D receptor).	Maximizes CTL infiltration, as well as the penetration and effectiveness of GEM and anti-PD-1.	(115)
Neutralization of Oncogenic Cargo	Targeting exosomal miR-21 (from PSCs/CAFs)	Preclinical (*In vitro*) and Clinical Data Analysis	Akt and Ras/ERK pathways are activated in cancer cells by miR-21.	Fosters cancer cell migration, EMT, and MMPs activity. One tactic may be to neutralize the other.	(116)
Neutralization of Oncogenic Cargo	Targeting exosomal Leptin/miR-224-3p/ABL2 axis	Preclinical (*In vitro* and *In vivo*)	Oncoprotein ABL2 is upregulated in cancer cells due to CAF-exosomal Leptin’s downregulation of miR-224-3p.	Motivates movement, invasion, and proliferation. Tumor promotion is attenuated by inhibition.	(117)
Neutralization of Oncogenic Cargo	Targeting exosomal miR-125b-5p	Preclinical (*In vitro*)	Targeting and inhibiting the tumor suppressor APC is the function of miR-125b-5p.	Supports cell growth, endothelial migration, and metastasis.	(118)
Neutralization of Oncogenic Cargo	Targeting exosomal miR-421/SIRT3/HIF-1α axis	Preclinical (*In vitro* and *In vivo*)	The transcription of H3K9Ac and HIF-1α is increased by miR-421, but SIRT3 is suppressed.	Boosts glycolysis, cell division, migration, and invasion. Tumor development is reduced by knocking it down.	(119)
Neutralization of Oncogenic Cargo	Engineered IEVs (Integrin α5-targeting EVs) loaded with PFD and miR-138-5p	Preclinical (*In vitro* and *In vivo*)	The miR-138-5p pathway blocks collagen production and TGF-β signaling in CAFs.	It reprograms CAFs, decreases fibrosis, increases chemosensitivity, alleviates hypoxia, and promotes drug perfusion.	(120)
Neutralization of Oncogenic Cargo	Targeting stromal miR-21 and miR-221	Preclinical (*In vitro*)	Stromal miRNAs enhance the clonogenicity and stemness of cancer cells. NF-κB/K-Ras is inhibited in CAFs by miR-221.	Sterilization inhibits tumor promotion and stromal invasiveness.	(121)
Neutralization of Oncogenic Cargo	Targeting exosomal miR-331-3p/SCARA5/FAK axis	Preclinical (*In vitro* and *In vivo*)	By binding to tumor suppressor SCARA5, miR-331-3p activates FAK signaling.	Helps cells divide, migrate, invade, and form tumors.	(122)
Neutralization of Oncogenic Cargo	Engineered ITGA5-EVs-148a (bone marrow MSC-EVs targeting Integrin α5, loaded with miR-148a-3p)	Preclinical (*In vitro* and *In vivo*)	Inhibits the TGF-β/SMAD pathway via ITGA5 by targeting CAFs.	Blocks tumor development, inactivates CAFs, and reverses tumor-promoting actions.	(123)
Oncogenic Cargo from Tumor to CAF	Targeting KRASG12D protein in CAFs (via tumor-derived EVs)	Preclinical (*In vitro* and *In vivo*)	The pro-tumorigenic and chemoresistance-promoting activities of CAFs are enhanced when tumor EVs transmit KRASG12D to them.	Chemoresistance may be reversed by MRTX1133 inhibition. Explains the poor prognosis of KRAS-mutant patients post-chemo.	(124)
Pathway Redundancy and Clinical Translation	Targeting IL-1/JAK/STAT3 pathway (Anakinra, Ruxolitinib)	Review and Clinical Trial Summary	Blocking inflammatory signaling in iCAFs lowers fibrosis and inflammation and enhances T cell infiltration.	*In vitro*, encouraging. Clinical studies in phase III were unsuccessful overall, but benefits were observed only in a subgroup of patients with significant inflammation.	(125–127)
CAF Heterogeneity and Precision Targeting	Targeting CXCR2/CXCL or CXCL12/CXCR4/CXCR7 axes	Review	Restricts the recruitment and exclusion of immune cells as well as paracrine CAF-tumor communication.	It can reduce tumor growth and increase survival in a preclinical setting. The need for precise regulation is highlighted by the fact that complete genetic ablation can sometimes accelerate malignancy.	(130)
CAF Heterogeneity and Precision Targeting	Selective CAF Depletion/Modulation, Such as CellaxTM-DTX NPs	Review	Focuses on certain subpopulations of CAFs rather than the whole stroma.	Try not to cause severe tumor development that happens with non-specific depletion.	(131)

## 7 Future directions

Even though there is significant hope for oncology with EXO-based treatments, their clinical translation is still in its infancy. The two primary methods now under consideration are 1) blocking the formation, release, or absorption of tumor-promoting EXOs and 2) using bioengineered EXOs as specific vectors for the delivery of medications, genes, or immunomodulators. The clinical effectiveness and safety of these methods are largely untested, despite promising anticancer potential in preclinical investigations. The majority of the data originates from animal and cell models. To develop therapeutically feasible targeted therapeutics based on EXOs, future investigations should focus on validating these targets in human trials and leveraging current mechanistic evidence (135).

There is great hope for overcoming resistance to cancer treatment using CAF-EVs. They can serve as non-invasive biomarkers for liquid biopsy and be engineered as targeted drug delivery systems. Their clinical translation, however, is impeded by significant obstacles. First, due to subjective approaches and isolation issues, there is no uniform, standardized framework for categorizing CAF subtypes. The second issue is the absence of standardized, good manufacturing practice-compliant techniques for separating and purifying EV subsets derived from CAF, as well as a mechanism for universally classifying these methods. Lastly, several challenges with current approaches to inhibiting tumor-promoting CAF-EV biogenesis and release include off-target effects, safety concerns (such as imipramine’s side effects), and the risk of activating compensatory pathways. Nevertheless, this strategy shows promise as a treatment. To effectively harness the therapeutic potential of CAF-EVs, we need to address these constraints by developing safer, more targeted approaches, more accurate isolation methods, and defined criteria (136).

This review has shown the significance of CAFs in PDAC tumors, as well as the complexity and apparent contradiction in their roles. It is for this reason that we would like to underscore the need for further advanced investigations focusing on the cellular origins of CAFs, how these precursors contribute to the CAF population, and how this contributes to stromal heterogeneity in PDAC. We also need markers that can reliably identify distinct CAF subsets and their potential functions, potentially serving as biomarkers to select for (new) targeted therapy approaches. It could therefore be helpful to explore and better understand the progressive changes in the stromal profile during PDAC development. To advance this subject, we believe more sophisticated ex vivo models should be used in conjunction with lineage tracing and scRNA-seq technologies. Due to technical constraints, this combination may not be practicable at present, but this does not diminish the need to create and implement such platforms in the near future. In conclusion, we feel that adequately modeling PDAC and deciphering the paradigm underlying CAF heterogeneity are critical for achieving clinically meaningful results and advancing targeted or customized therapy (137).

## 8 Conclusion

This research reviewed that CAFs and the EXOs they produce are master orchestrators of PC’s aggressive and treatment-resistant milieu. Researchers have made considerable progress—identifying multiple CAF subtypes, documenting the chemical cargo in their EXOs, and correlating them to tumor growth, dissemination, immunological evasion, and treatment resistance. Yet despite these breakthroughs, translating these insights into viable medicines for patients remains a major challenge.

Numerous key challenges stand in the way. First, CAFs aren’t a single enemy—some actually assist in limiting tumors, and we still don’t know how to attack the “bad” ones without injuring the “good.” Second, the majority of studies use laboratory models that are too simplistic to accurately reflect the intricacy of actual cancers. Third, studies tend to focus on a single exosomal molecule at a time, omitting the broader picture of how various signals function together. Lastly, there is a significant gap between promising laboratory discoveries and practical treatments due to challenges such as large-scale production of medicines, separating certain types of EXOs from blood, and ensuring the safety of these methods.

The discipline requires a paradigm change toward precision stromal medicine to overcome these obstacles. This involves using cutting-edge technology to track EXO signals and CAF subtypes in specific individuals over time. We need more accurate disease-mimicking laboratory models, such as patient-derived organoids. Smart NPs or modified EXO decoys could be used to target several EXO signals concurrently. These therapies should then be deliberately integrated with existing immunotherapy and chemotherapy. Big, biomarker-guided clinical trials and universally accepted criteria are essential for this to succeed.

The original plan to only eliminate the stroma has obviously failed; that is no longer the objective. Instead, exactly reprogramming it is where the future is. We may increase survival rates for PC by understanding and disrupting the complex communication between CAFs and cancer cells, tailoring this approach to each patient. This will transform the disease’s strongest defense into its weakest point.
